# Supplementary material for: Biocatalysed synthesis planning using data-driven learning
Source: Nat Commun. 2022 Feb 18;13:964. doi: 10.1038/s41467-022-28536-w (PMC8857209; doi:10.1038/s41467-022-28536-w)
Supplement: Supplementary file 1 — Supplementary Information [file 41467_2022_28536_MOESM1_ESM.pdf]

Supplementary Information: Biocatalysed Synthesis Planning using Data-driven Learning

Daniel Probst[1,2,*], Matteo Manica[1], Yves Gaëtan Nana Teukam[1], Alessandro Castrogiovanni[1,2], Federico Paratore[1], and Teodoro Laino[1,2]

[1]*IBM Research Europe, CH-8803 Rüschlikon, Switzerland*
[*]*dpr@zurich.ibm.com*
[2]*National Center for Competence in Research-Catalysis (NCCR-Catalysis), Switzerland*

Supplementary Discussion

Additional Figures

Name	SMARTS
Coenzyme A	O=C(NCC*)CCNC(=O)C(O)C(C)(C)COP(=O)(*)OP(=O)(*)OC*3O*(n2cnc1c(ncnc12)N)*(O)*3OP(=O)(*)*
Nicotinamide adenine dinucleotides	**1*(*)*(COP(*)(=O)OP(*)(=O)OC*2O*(*)*(*)*2*)O*1*
Nucleoside phosphates	**1*(*)*(O*1COP(*)(=O)O)[R]
Nucleoside phosphates isomers	*P(*)(=O)O*1*(*)*(*)O[*]1COP(*)(*)=O
Sulfonium betaines	**1*(*)*(O*1CS*)[R]
Flavines	**1**2**3*(**(=O)**3=O)*(*)*2**1*
Hemes	*~1~*~*~2~*~*~1~*~*~1~*~*~*(~*~*~3~*~*~*(~*~*~4~*~*~*(~*~2)~*~4)~*~3)~*~1
Iron-sulfur cluster(s)	S1[Fe]S[Fe]1

Supplementary Table 1: SMARTS patterns of co-enzymes that were removed from the products.

Name	SMILES
Phosphate trianion	O=P([O-])([O-])[O-]
Hydrogen phosphate dianion	O=P([O-])([O-])O
(2-hydroxyethyl)trimethylammonium	C[N+](C)(C)CCO
Ethanolamine	NCCO
Diphosphate	O=P([O-])([O-])OP(=O)([O-])[O-]
Hydrogen diphosphate trianion	O=P([O-])([O-])OP(=O)([O-])O
2-Oxoglutarate dianion	O=C([O-])CCC(=O)C(=O)[O-]
Acetate ion	CC(=O)[O-]
Pyruvate	CC(=O)C(=O)[O-]

Supplementary Table 2: SMILES of common byproducts that were removed from the products.

EC number	Count	% (of total)	EC number	Count	% (of total)	EC number	Count	% (of total)	EC number	Count	% (of total)
1.-.x.x	75	0.120	1.7.x.x	191	0.306	3.2.x.x	888	1.423	5.2.x.x	36	0.058
1.1.x.x	4481	7.181	1.8.x.x	248	0.397	3.3.x.x	148	0.237	5.3.x.x	451	0.723
1.10.x.x	80	0.128	1.9.x.x	16	0.026	3.4.x.x	419	0.671	5.4.x.x	360	0.577
1.11.x.x	191	0.306	1.97.x.x	19	0.030	3.5.x.x	1082	1.734	5.5.x.x	198	0.317
1.12.x.x	34	0.054	2.-.x.x	8	0.013	3.6.x.x	1372	2.199	5.6.x.x	8	0.013
1.13.x.x	665	1.066	2.1.x.x	4420	7.083	3.7.x.x	115	0.184	5.99.x.x	5	0.008
1.14.x.x	4800	7.692	2.10.x.x	3	0.005	3.8.x.x	109	0.175	6.-.x.x	4	0.006
1.16.x.x	43	0.069	2.2.x.x	55	0.088	3.9.x.x	14	0.022	6.1.x.x	158	0.253
1.17.x.x	201	0.322	2.3.x.x	10369	16.616	3.A.x.x	21	0.034	6.2.x.x	517	0.828
1.18.x.x	43	0.069	2.4.x.x	2015	3.229	4.-.x.x	6	0.010	6.3.x.x	534	0.856
1.19.x.x	11	0.018	2.5.x.x	580	0.929	4.1.x.x	2024	3.243	6.4.x.x	47	0.075
1.2.x.x	1404	2.250	2.6.x.x	175	0.280	4.2.x.x	1762	2.824	6.5.x.x	26	0.042
1.20.x.x	19	0.030	2.7.x.x	14973	23.994	4.3.x.x	188	0.301	6.6.x.x	5	0.008
1.21.x.x	65	0.104	2.8.x.x	419	0.671	4.4.x.x	196	0.314	7.1.x.x	33	0.053
1.22.x.x	3	0.005	2.9.x.x	10	0.016	4.5.x.x	19	0.030	7.2.x.x	56	0.090
1.23.x.x	13	0.021	3.-.x.x	6	0.010	4.6.x.x	39	0.062	7.3.x.x	17	0.027
1.3.x.x	1362	2.183	3.1.x.x	2861	4.585	4.7.x.x	2	0.003	7.4.x.x	45	0.072
1.4.x.x	443	0.710	3.10.x.x	3	0.005	4.99.x.x	65	0.104	7.5.x.x	36	0.058
1.5.x.x	376	0.603	3.11.x.x	4	0.006	5.-.x.x	9	0.014	7.6.x.x	66	0.106
1.6.x.x	260	0.417	3.13.x.x	20	0.032	5.1.x.x	359	0.575			

Supplementary Table 3: The data set composition by EC-level 2.

EC number	Count	% (of total)	EC number	Count	% (of total)	EC number	Count	% (of total)	EC number	Count	% (of total)
1.-.-.x	75	0.120	1.21.1.x	12	0.019	2.4.99.x	92	0.147	3.7.1.x	115	0.184
1.1.-.x	30	0.048	1.21.21.x	1	0.002	2.5.1.x	580	0.929	3.8.1.x	109	0.175
1.1.1.x	4023	6.447	1.21.3.x	35	0.056	2.6.-.x	1	0.002	3.9.1.x	14	0.022
1.1.2.x	45	0.072	1.21.4.x	2	0.003	2.6.1.x	167	0.268	3.A.1.x	19	0.030
1.1.3.x	245	0.393	1.21.98.x	6	0.010	2.6.99.x	7	0.011	3.A.3.x	2	0.003
1.1.4.x	1	0.002	1.21.99.x	8	0.013	2.7.-.x	2	0.003	4.-.-.x	6	0.010
1.1.5.x	33	0.053	1.22.1.x	3	0.005	2.7.1.x	1187	1.902	4.1.-.x	1	0.002
1.1.7.x	1	0.002	1.23.1.x	11	0.018	2.7.10.x	5	0.008	4.1.1.x	1594	2.554
1.1.9.x	1	0.002	1.23.5.x	2	0.003	2.7.11.x	31	0.050	4.1.2.x	214	0.343
1.1.98.x	18	0.029	1.3.-.x	20	0.032	2.7.12.x	10	0.016	4.1.3.x	107	0.171
1.1.99.x	84	0.135	1.3.1.x	936	1.500	2.7.13.x	7	0.011	4.1.4.x	1	0.002
1.10.1.x	1	0.002	1.3.2.x	18	0.029	2.7.14.x	3	0.005	4.1.99.x	107	0.171
1.10.2.x	7	0.011	1.3.3.x	121	0.194	2.7.2.x	77	0.123	4.2.1.x	996	1.596
1.10.3.x	68	0.109	1.3.5.x	11	0.018	2.7.3.x	29	0.046	4.2.2.x	41	0.066
1.10.5.x	3	0.005	1.3.7.x	40	0.064	2.7.4.x	163	0.261	4.2.3.x	701	1.123
1.10.99.x	1	0.002	1.3.8.x	134	0.215	2.7.6.x	29	0.046	4.2.99.x	24	0.038
1.11.-.x	4	0.006	1.3.98.x	17	0.027	2.7.7.x	766	1.228	4.3.-.x	2	0.003
1.11.1.x	135	0.216	1.3.99.x	65	0.104	2.7.8.x	12645	20.263	4.3.1.x	119	0.191
1.11.2.x	52	0.083	1.4.-.x	3	0.005	2.7.9.x	18	0.029	4.3.2.x	33	0.053
1.12.1.x	12	0.019	1.4.1.x	138	0.221	2.7.99.x	1	0.002	4.3.3.x	26	0.042
1.12.2.x	2	0.003	1.4.13.x	4	0.006	2.8.1.x	39	0.062	4.3.99.x	8	0.013
1.12.5.x	3	0.005	1.4.2.x	6	0.010	2.8.2.x	225	0.361	4.4.1.x	196	0.314
1.12.7.x	2	0.003	1.4.3.x	250	0.401	2.8.3.x	141	0.226	4.5.1.x	19	0.030
1.12.98.x	11	0.018	1.4.4.x	3	0.005	2.8.4.x	7	0.011	4.6.1.x	39	0.062
1.12.99.x	4	0.006	1.4.5.x	6	0.010	2.8.5.x	7	0.011	4.7.1.x	2	0.003
1.13.-.x	7	0.011	1.4.7.x	5	0.008	2.9.1.x	10	0.016	4.99.1.x	65	0.104
1.13.11.x	527	0.845	1.4.9.x	3	0.005	3.-.-.x	6	0.010	5.-.-.x	9	0.014
1.13.12.x	115	0.184	1.4.99.x	25	0.040	3.1.-.x	4	0.006	5.1.-.x	3	0.005
1.13.99.x	16	0.026	1.5.-.x	2	0.003	3.1.1.x	1006	1.612	5.1.1.x	124	0.199
1.14.-.x	118	0.189	1.5.1.x	258	0.413	3.1.11.x	6	0.010	5.1.2.x	23	0.037
1.14.11.x	291	0.466	1.5.3.x	64	0.103	3.1.13.x	5	0.008	5.1.3.x	182	0.292
1.14.12.x	191	0.306	1.5.5.x	7	0.011	3.1.14.x	2	0.003	5.1.99.x	27	0.043
1.14.13.x	1611	2.582	1.5.7.x	7	0.011	3.1.15.x	1	0.002	5.2.-.x	4	0.006
1.14.14.x	1312	2.102	1.5.8.x	10	0.016	3.1.2.x	326	0.522	5.2.1.x	32	0.051
1.14.15.x	338	0.542	1.5.99.x	28	0.045	3.1.21.x	6	0.010	5.3.-.x	1	0.002
1.14.16.x	2	0.003	1.6.-.x	1	0.002	3.1.22.x	1	0.002	5.3.1.x	208	0.333
1.14.17.x	7	0.011	1.6.1.x	2	0.003	3.1.26.x	6	0.010	5.3.2.x	35	0.056
1.14.18.x	141	0.226	1.6.2.x	25	0.040	3.1.27.x	8	0.013	5.3.3.x	155	0.248
1.14.19.x	442	0.708	1.6.3.x	26	0.042	3.1.3.x	1167	1.870	5.3.4.x	3	0.005
1.14.20.x	70	0.112	1.6.4.x	3	0.005	3.1.4.x	222	0.356	5.3.99.x	49	0.079
1.14.21.x	43	0.069	1.6.5.x	176	0.282	3.1.5.x	2	0.003	5.4.1.x	13	0.021
1.14.3.x	1	0.002	1.6.6.x	5	0.008	3.1.6.x	43	0.069	5.4.2.x	75	0.120
1.14.99.x	233	0.373	1.6.98.x	1	0.002	3.1.7.x	36	0.058	5.4.3.x	43	0.069
1.16.1.x	37	0.059	1.6.99.x	21	0.034	3.1.8.x	20	0.032	5.4.4.x	67	0.107
1.16.3.x	1	0.002	1.7.-.x	2	0.003	3.10.1.x	3	0.005	5.4.99.x	162	0.260
1.16.5.x	1	0.002	1.7.1.x	91	0.146	3.11.1.x	4	0.006	5.5.1.x	198	0.317
1.16.8.x	3	0.005	1.7.2.x	44	0.071	3.13.1.x	20	0.032	5.6.1.x	8	0.013
1.16.9.x	1	0.002	1.7.3.x	29	0.046	3.2.-.x	1	0.002	5.99.-.x	1	0.002
1.17.-.x	2	0.003	1.7.5.x	6	0.010	3.2.1.x	803	1.287	5.99.1.x	4	0.006
1.17.1.x	69	0.111	1.7.6.x	2	0.003	3.2.2.x	84	0.135	6.-.-.x	4	0.006
1.17.2.x	8	0.013	1.7.7.x	9	0.014	3.3.1.x	10	0.016	6.1.-.x	2	0.003
1.17.3.x	47	0.075	1.7.99.x	8	0.013	3.3.2.x	138	0.221	6.1.1.x	142	0.228
1.17.4.x	25	0.040	1.8.1.x	137	0.220	3.4.-.x	9	0.014	6.1.2.x	7	0.011
1.17.5.x	13	0.021	1.8.2.x	26	0.042	3.4.11.x	108	0.173	6.1.3.x	7	0.011
1.17.7.x	16	0.026	1.8.3.x	30	0.048	3.4.13.x	80	0.128	6.2.1.x	516	0.827
1.17.8.x	4	0.006	1.8.4.x	16	0.026	3.4.14.x	12	0.019	6.2.2.x	1	0.002
1.17.9.x	2	0.003	1.8.5.x	17	0.027	3.4.15.x	6	0.010	6.3.-.x	2	0.003
1.17.98.x	6	0.010	1.8.7.x	9	0.014	3.4.16.x	28	0.045	6.3.1.x	69	0.111
1.17.99.x	9	0.014	1.8.98.x	6	0.010	3.4.17.x	42	0.067	6.3.2.x	306	0.490
1.18.-.x	1	0.002	1.8.99.x	7	0.011	3.4.18.x	1	0.002	6.3.3.x	28	0.045
1.18.1.x	32	0.051	1.9.3.x	7	0.011	3.4.19.x	50	0.080	6.3.4.x	84	0.135
1.18.4.x	1	0.002	1.9.6.x	4	0.006	3.4.21.x	29	0.046	6.3.5.x	45	0.072
1.18.6.x	5	0.008	1.9.98.x	3	0.005	3.4.22.x	18	0.029	6.4.1.x	47	0.075
1.18.99.x	4	0.006	1.9.99.x	2	0.003	3.4.23.x	7	0.011	6.5.-.x	1	0.002
1.19.1.x	8	0.013	1.97.1.x	19	0.030	3.4.24.x	21	0.034	6.5.1.x	25	0.040
1.19.6.x	3	0.005	2.-.-.x	8	0.013	3.4.25.x	6	0.010	6.6.1.x	5	0.008
1.2.-.x	5	0.008	2.1.1.x	4352	6.974	3.4.99.x	2	0.003	7.1.1.x	25	0.040
1.2.1.x	1157	1.854	2.1.2.x	28	0.045	3.5.-.x	1	0.002	7.1.2.x	4	0.006
1.2.2.x	9	0.014	2.1.3.x	38	0.061	3.5.1.x	606	0.971	7.1.3.x	4	0.006
1.2.3.x	94	0.151	2.1.4.x	1	0.002	3.5.2.x	91	0.146	7.2.1.x	8	0.013
1.2.4.x	51	0.082	2.1.5.x	1	0.002	3.5.3.x	73	0.117	7.2.2.x	36	0.058
1.2.5.x	27	0.043	2.10.1.x	3	0.005	3.5.4.x	161	0.258	7.2.4.x	12	0.019
1.2.7.x	48	0.077	2.2.1.x	55	0.088	3.5.5.x	96	0.154	7.3.2.x	17	0.027
1.2.98.x	4	0.006	2.3.-.x	4	0.006	3.5.99.x	54	0.087	7.4.2.x	45	0.072
1.2.99.x	9	0.014	2.3.1.x	10174	16.304	3.6.-.x	1	0.002	7.5.2.x	36	0.058
1.20.1.x	9	0.014	2.3.2.x	101	0.162	3.6.1.x	551	0.883	7.6.1.x	1	0.002
1.20.2.x	4	0.006	2.3.3.x	90	0.144	3.6.2.x	6	0.010	7.6.2.x	65	0.104
1.20.4.x	2	0.003	2.4.-.x	5	0.008	3.6.3.x	794	1.272			
1.20.9.x	4	0.006	2.4.1.x	1732	2.776	3.6.4.x	16	0.026			
1.21.-.x	1	0.002	2.4.2.x	186	0.298	3.6.5.x	4	0.006			

Supplementary Table 4: The data set composition by EC-level 3.

	**n = 1**		**n < 5**		**n < 10**		**n < 100**		**n ≥ 100**		**Total**
	count	%	count	%	count	%	count	%	count	%	count
**EC1**	0	0.0	0	0.0	0	0.0	0	0.0	7	100	7
**EC2**	2	2.6	7	9.0	12	15.4	42	53.8	36	46.2	78
**EC3**	33	10.7	81	26.2	129	41.7	248	80.3	61	19.7	309
**EC4**	1593	25.3	4473	71.1	5571	88.6	6251	99.4	38	0.6	6460

Supplementary Table 5: The data set composition by size n of sub set and EC level.

EC number	Train Samples	Test Samples
1.-.x.x	58	6
1.1.x.x	3569	161
1.10.x.x	66	4
1.11.x.x	148	7
1.12.x.x	26	1
1.13.x.x	419	65
1.14.x.x	3793	259
1.16.x.x	42	0
1.17.x.x	167	6
1.18.x.x	44	0
1.19.x.x	10	0
1.2.x.x	1126	31
1.20.x.x	15	0
1.21.x.x	48	7
1.23.x.x	10	2
1.3.x.x	1077	60
1.4.x.x	332	18
1.5.x.x	280	13
1.6.x.x	216	5
1.7.x.x	186	6
1.8.x.x	209	7
1.9.x.x	12	1
1.97.x.x	17	0
2.-.x.x	8	0
2.1.x.x	3759	184
2.10.x.x	3	0
2.2.x.x	48	1
2.3.x.x	9041	567
2.4.x.x	1570	79
2.5.x.x	577	20
2.6.x.x	129	4
2.7.x.x	13217	726
2.8.x.x	338	13
2.9.x.x	9	0
3.-.x.x	5	0
3.1.x.x	2092	149
3.10.x.x	1	0
3.11.x.x	1	0
3.13.x.x	10	2
3.2.x.x	387	17
3.3.x.x	106	12
3.4.x.x	151	8
3.5.x.x	694	51
3.6.x.x	1207	14
3.7.x.x	53	3
3.8.x.x	75	11
3.9.x.x	10	0
3.A.x.x	19	0
4.-.x.x	6	0
4.1.x.x	1640	50
4.2.x.x	1201	109
4.3.x.x	119	8
4.4.x.x	142	7
4.5.x.x	10	1
4.6.x.x	22	1
4.7.x.x	2	0
4.99.x.x	46	1
5.-.x.x	5	0
5.1.x.x	264	13
5.2.x.x	28	0
5.3.x.x	341	21
5.4.x.x	252	17
5.5.x.x	159	8
5.6.x.x	6	0
5.99.x.x	2	0
6.-.x.x	4	0
6.1.x.x	101	1
6.2.x.x	416	6
6.3.x.x	527	54
6.4.x.x	37	0
6.5.x.x	25	1
6.6.x.x	4	0
7.1.x.x	30	1
7.2.x.x	36	0
7.3.x.x	8	0
7.4.x.x	32	0
7.5.x.x	30	0
7.6.x.x	55	0

Supplementary Table 6: The data set composition after train/test split at EC-level 2.

EC number	Train Samples	Test Samples
1.-.-.x	58	6
1.1.-.x	25	0
1.1.1.x	3220	141
1.1.2.x	40	0
1.1.3.x	176	15
1.1.4.x	1	0
1.1.5.x	33	0
1.1.7.x	1	0
1.1.9.x	1	0
1.1.98.x	7	0
1.1.99.x	65	5
1.10.2.x	8	0
1.10.3.x	56	4
1.10.5.x	2	0
1.11.-.x	3	0
1.11.1.x	104	3
1.11.2.x	41	4
1.12.1.x	10	0
1.12.2.x	1	0
1.12.5.x	2	0
1.12.7.x	2	0
1.12.98.x	8	1
1.12.99.x	3	0
1.13.-.x	3	1
1.13.11.x	339	58
1.13.12.x	64	5
1.13.99.x	13	1
1.14.-.x	99	9
1.14.11.x	55	3
1.14.12.x	150	10
1.14.13.x	1329	94
1.14.14.x	1123	85
1.14.15.x	303	12
1.14.16.x	2	0
1.14.17.x	7	1
1.14.18.x	124	5
1.14.19.x	375	22
1.14.20.x	3	0
1.14.21.x	36	2
1.14.3.x	1	0
1.14.99.x	186	16
1.16.1.x	37	0
1.16.3.x	1	0
1.16.5.x	1	0
1.16.8.x	2	0
1.16.9.x	1	0
1.17.-.x	2	0
1.17.1.x	58	1
1.17.2.x	8	0
1.17.3.x	36	2
1.17.4.x	22	0
1.17.5.x	13	1
1.17.7.x	13	0
1.17.8.x	3	0
1.17.9.x	1	0
1.17.98.x	5	0
1.17.99.x	6	2
1.18.-.x	1	0
1.18.1.x	32	0
1.18.4.x	1	0
1.18.6.x	6	0
1.18.99.x	4	0
1.19.1.x	8	0
1.19.6.x	2	0
1.2.-.x	5	0
1.2.1.x	921	27
1.2.2.x	8	0
1.2.3.x	69	1
1.2.4.x	47	2
1.2.5.x	22	0
1.2.7.x	45	0
1.2.98.x	1	1
1.2.99.x	8	0
1.20.1.x	7	0
1.20.2.x	4	0
1.20.4.x	2	0
1.20.9.x	2	0
1.21.-.x	1	0
1.21.1.x	10	0
1.21.21.x	1	0
1.21.3.x	25	3
1.21.4.x	2	0
1.21.98.x	3	2
1.21.99.x	6	2
1.23.1.x	9	2
1.23.5.x	1	0
1.3.-.x	18	1
1.3.1.x	718	46
1.3.2.x	15	2
1.3.3.x	91	6
1.3.5.x	10	0
1.3.7.x	44	1
1.3.8.x	120	3
1.3.98.x	9	0
1.3.99.x	52	1
1.4.-.x	3	0
1.4.1.x	101	3
1.4.13.x	2	2
1.4.2.x	6	0
1.4.3.x	182	12
1.4.4.x	5	1
1.4.5.x	6	0
1.4.7.x	4	0
1.4.9.x	1	0
1.4.99.x	22	0
1.5.-.x	2	0
1.5.1.x	202	12
1.5.3.x	32	1
1.5.5.x	7	0
1.5.7.x	6	0
1.5.8.x	10	0
1.5.99.x	21	0
1.6.-.x	1	0
1.6.1.x	2	0
1.6.2.x	23	1
1.6.3.x	21	1
1.6.4.x	2	0
1.6.5.x	150	3
1.6.6.x	1	0
1.6.98.x	1	0
1.6.99.x	15	0
1.7.-.x	2	0
1.7.1.x	70	5
1.7.2.x	48	1
1.7.3.x	23	0
1.7.5.x	16	0
1.7.6.x	2	0
1.7.7.x	10	0
1.7.99.x	15	0
1.8.1.x	117	4
1.8.2.x	21	0
1.8.3.x	18	2
1.8.4.x	10	0
1.8.5.x	17	1
1.8.7.x	12	0
1.8.98.x	8	0
1.8.99.x	6	0
1.9.3.x	4	0
1.9.6.x	4	1
1.9.98.x	3	0
1.9.99.x	1	0
1.97.1.x	17	0
2.-.-.x	8	0
2.1.1.x	3698	183
2.1.2.x	24	0
2.1.3.x	35	1
2.1.4.x	1	0
2.1.5.x	1	0
2.10.1.x	3	0
2.2.1.x	48	1
2.3.-.x	4	0
2.3.1.x	8887	561
2.3.2.x	84	4
2.3.3.x	66	2
2.4.-.x	4	0
2.4.1.x	1358	69
2.4.2.x	141	6
2.4.99.x	67	4
2.5.1.x	577	20
2.6.1.x	122	3
2.6.3.x	2	0
2.6.99.x	5	1
2.7.-.x	2	0
2.7.1.x	964	10
2.7.10.x	5	0
2.7.11.x	21	2
2.7.12.x	8	0
2.7.13.x	7	0
2.7.14.x	3	0
2.7.2.x	60	0
2.7.3.x	22	1
2.7.4.x	135	2
2.7.6.x	23	0
2.7.7.x	669	5
2.7.8.x	11278	706
2.7.9.x	19	0
2.7.99.x	1	0
2.8.1.x	38	1
2.8.2.x	179	12
2.8.3.x	108	0
2.8.4.x	7	0
2.8.5.x	6	0
2.9.1.x	9	0
3.-.-.x	5	0
3.1.-.x	1	1
3.1.1.x	746	46
3.1.11.x	9	0
3.1.12.x	1	0
3.1.13.x	4	1
3.1.14.x	1	0
3.1.15.x	1	0
3.1.16.x	2	0
3.1.2.x	223	15
3.1.21.x	5	0
3.1.26.x	5	0
3.1.27.x	8	0
3.1.3.x	951	73
3.1.4.x	104	8
3.1.6.x	7	0
3.1.7.x	20	4
3.1.8.x	4	1
3.10.1.x	1	0
3.11.1.x	1	0
3.13.1.x	10	2
3.2.1.x	358	12
3.2.2.x	29	5
3.3.1.x	6	0
3.3.2.x	100	12
3.4.-.x	5	0
3.4.11.x	43	1
3.4.13.x	17	0
3.4.14.x	4	1
3.4.15.x	3	2
3.4.16.x	10	0
3.4.17.x	12	1
3.4.19.x	13	1
3.4.21.x	18	1
3.4.22.x	5	1
3.4.23.x	6	0
3.4.24.x	10	0
3.4.25.x	5	0
3.5.-.x	1	0
3.5.1.x	378	24
3.5.2.x	59	5
3.5.3.x	31	2
3.5.4.x	105	15
3.5.5.x	83	3
3.5.99.x	37	2
3.6.1.x	440	13
3.6.2.x	2	0
3.6.3.x	751	0
3.6.4.x	11	1
3.6.5.x	3	0
3.7.1.x	53	3
3.8.1.x	75	11
3.9.1.x	10	0
3.A.1.x	17	0
3.A.3.x	2	0
4.-.-.x	6	0
4.1.-.x	1	0
4.1.1.x	1359	38
4.1.2.x	134	7
4.1.3.x	64	2
4.1.99.x	82	3
4.2.1.x	731	45
4.2.2.x	25	3
4.2.3.x	435	60
4.2.99.x	10	1
4.3.-.x	1	0
4.3.1.x	79	3
4.3.2.x	18	1
4.3.3.x	17	4
4.3.99.x	4	0
4.4.1.x	142	7
4.5.1.x	10	1
4.6.1.x	22	1
4.7.1.x	2	0
4.99.1.x	46	1
5.-.-.x	5	0
5.1.-.x	3	0
5.1.1.x	86	7
5.1.2.x	15	1
5.1.3.x	140	5
5.1.99.x	20	0
5.2.-.x	4	0
5.2.1.x	24	0
5.3.-.x	1	0
5.3.1.x	168	5
5.3.2.x	21	2
5.3.3.x	113	9
5.3.4.x	3	0
5.3.99.x	35	5
5.4.1.x	10	0
5.4.2.x	57	3
5.4.3.x	29	3
5.4.4.x	52	2
5.4.99.x	104	9
5.5.1.x	159	8
5.6.1.x	6	0
5.99.1.x	2	0
6.-.-.x	4	0
6.1.-.x	1	0
6.1.1.x	89	0
6.1.2.x	5	1
6.1.3.x	6	0
6.2.1.x	416	6
6.3.-.x	22	3
6.3.1.x	58	5
6.3.2.x	325	45
6.3.3.x	22	0
6.3.4.x	69	1
6.3.5.x	31	0
6.4.1.x	37	0
6.5.1.x	25	1
6.6.1.x	4	0
7.1.1.x	21	1
7.1.2.x	4	0
7.1.3.x	5	0
7.2.1.x	7	0
7.2.2.x	22	0
7.2.4.x	7	0
7.3.2.x	8	0
7.4.2.x	32	0
7.5.2.x	30	0
7.6.1.x	1	0
7.6.2.x	54	0

Supplementary Table 7: The data set composition after train/test split at EC-level 3.

1 EC-level 1 analysis

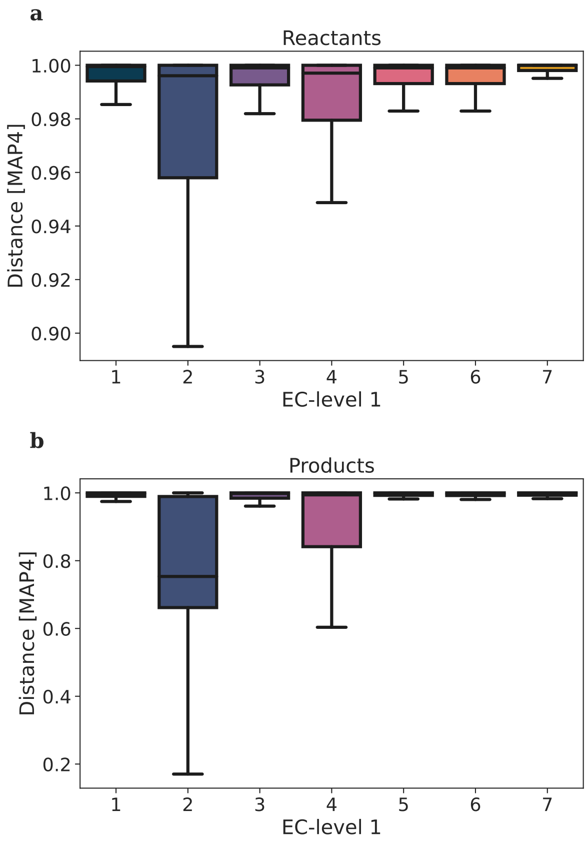

Supplementary Figure 1: Sampled (10%) intra-class MAP4 distances of unique reactants (**a**) and products (**b**) participating in reactions in EC3. Transferases (2), lyases (4), and to a lesser extent hydrolases (3) show lower mean distances compared to other classes. This confirms the findings of the visual inspection carried out on the TMAP in Figure 2b and c. The existence of homogeneous clusters of molecules within a class acts as an implicit feature, reducing the importance of the EC number token (explicit feature) during training and might increase accuracy compared to other classes.

2 EC-level 2 analysis

The most represented subclasses at EC-level 2 are EC 2.7.x.x (transferases transferring phosphorus-containing groups) with 24.5%, EC 2.3.x.x (acetyltransferases) with 16.8%, EC 1.1.x.x (oxidoreductases acting on the CH-OH group of donors) with 8%, EC 2.1.x.x (transferases transferring one-carbon groups) with 7.5%, EC 1.14.x.x (oxidoreductases acting on paired donors, with incorporation or reduction of molecular oxygen) with 7.3%, EC 3.1.x.x (hydrolases acting on ester bonds) with 4.5%, EC 4.1.x.x (carbon-carbon lyases) with 3%, EC 2.4.x.x (glycosyltransferases) with 2.9%, EC 4.2.x.x (carbon-oxygen lyases) with 2.5%, and EC 3.6.x.x (hydrolases acting on acting on acid anhydrides) with 2.5%.

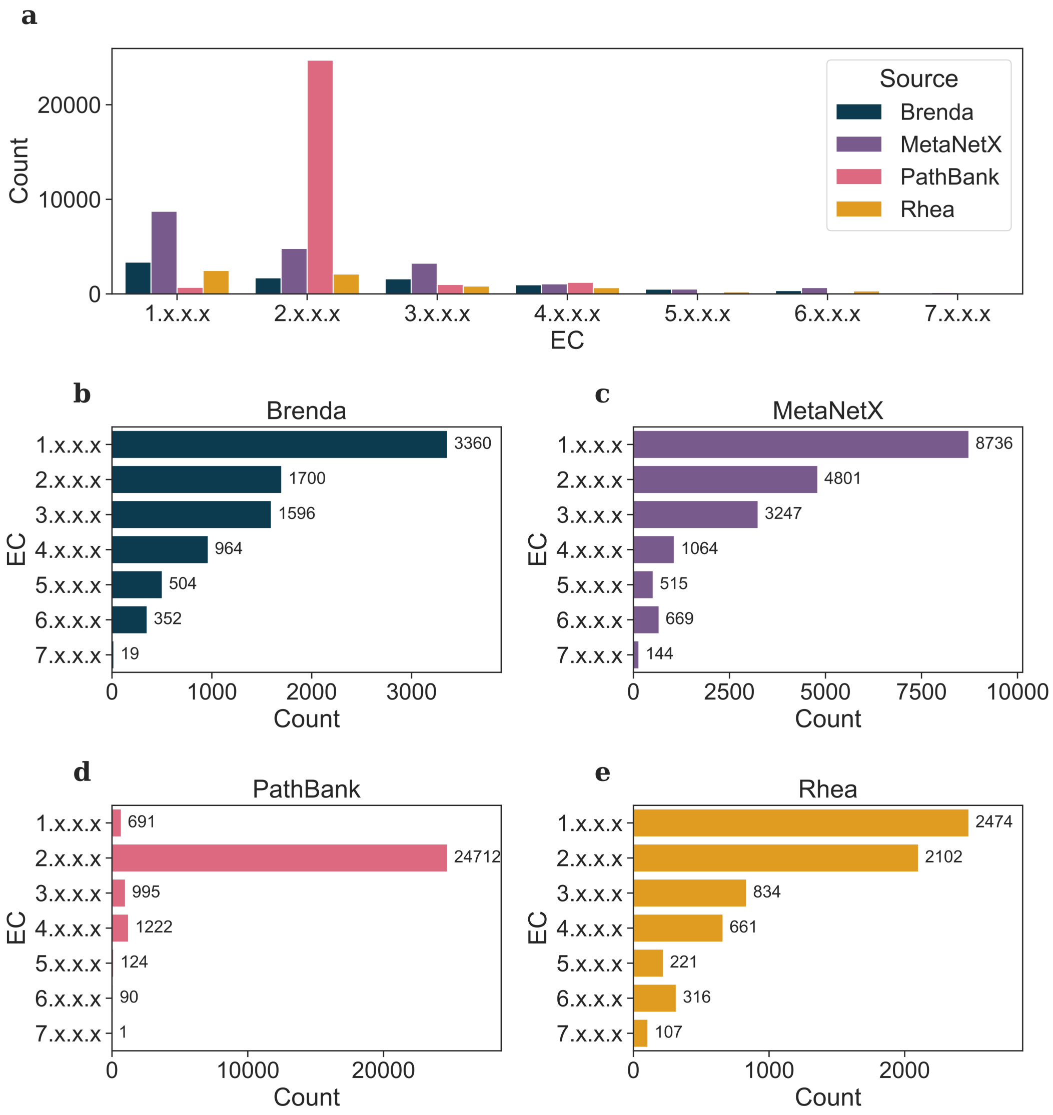

Supplementary Figure 2: Data sources for the ECREACT data set. (**a**) The overall composition of the data set by EC-level 1 and source. (**b-e**) Composition of the data by EC-level 1 imported from Brenda, MetaNetX, PathBank, and Rhea, respectively. The large number of transferase-catalysed reactions imported from PathBank reflects the high number of lipid pathways stored in the database.

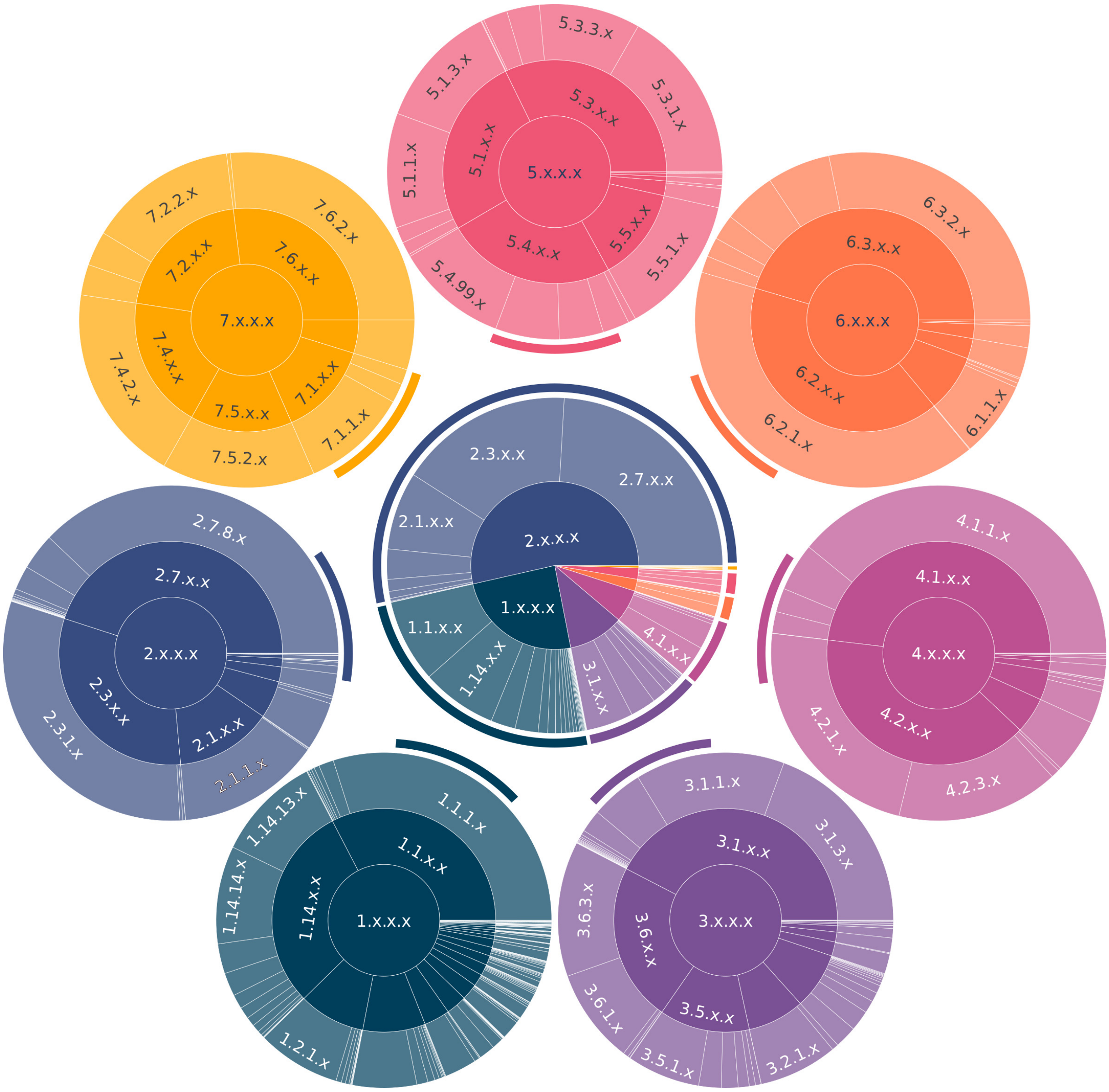

Supplementary Figure 3: The distribution of samples at EC-levels 1 (corresponding to enzyme classes) and 2, as well as EC-levels 2 and 3 of oxidoreductases (class 1), transferases (class 2), hydrolases (class 3), lyases (class 4), isomerases (class 5), ligases (class 6), and translocases (class 7), in the ECREACT EC3 data set.

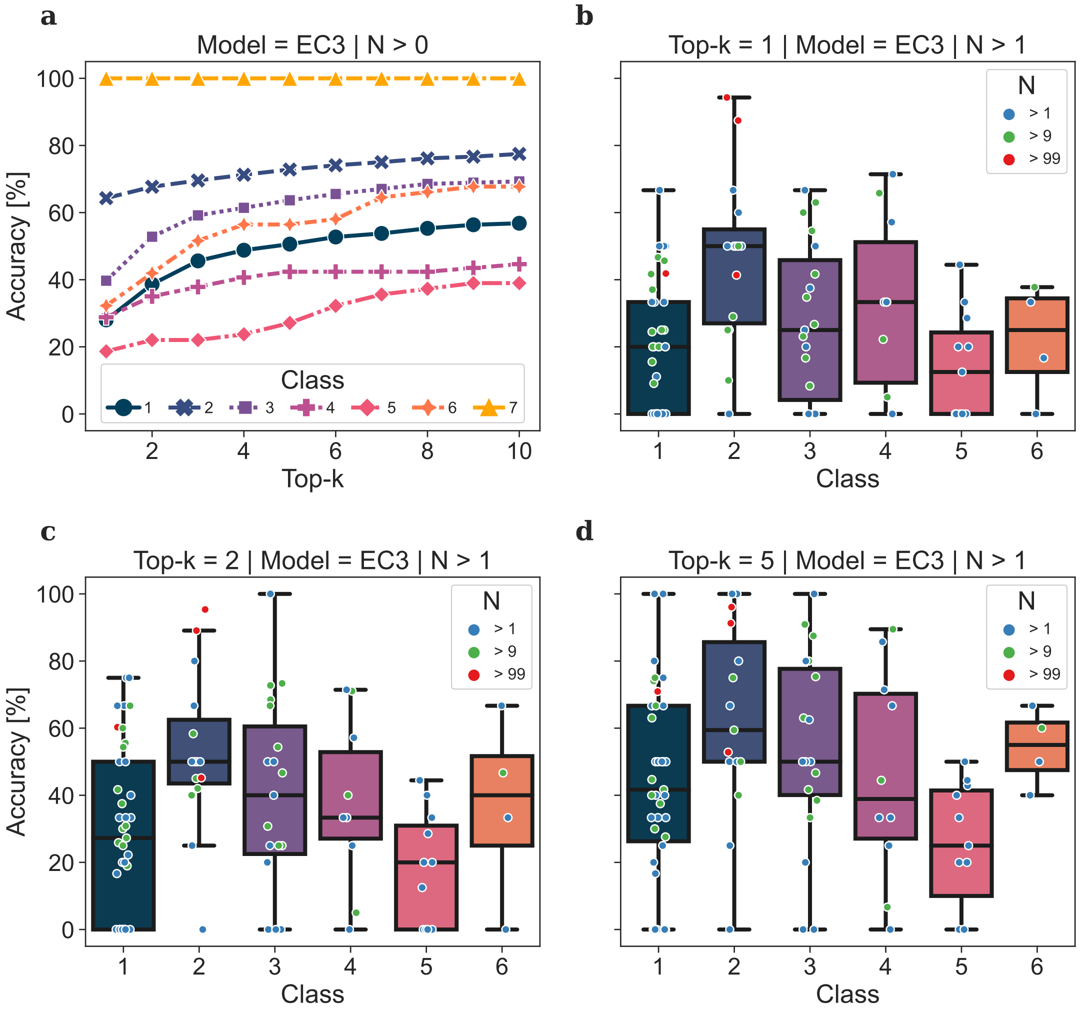

Supplementary Figure 4: Class-wise accuracy for the forward model trained on EC3. (**a**) The top-k prediction accuracies for each class show significant differences among classes caused by the number of available samples per EC-level 3 category. The accuracy of (**b**) top-1, (**c**) top-2, and (**d**) top-5 predictions per EC-level 3 category. Each dot represents an EC-level 3 subclass coloured by the number of test samples N. Large EC-level 3 subclasses (red) greatly influence the performance of predicting transferase-catalysed reaction (class 2) outcomes. Oxidoreductase-catalysed reactions (class 1) are distributed among many EC-level 3 subclasses, causing a lower performance compared to other classes with fewer samples overall.

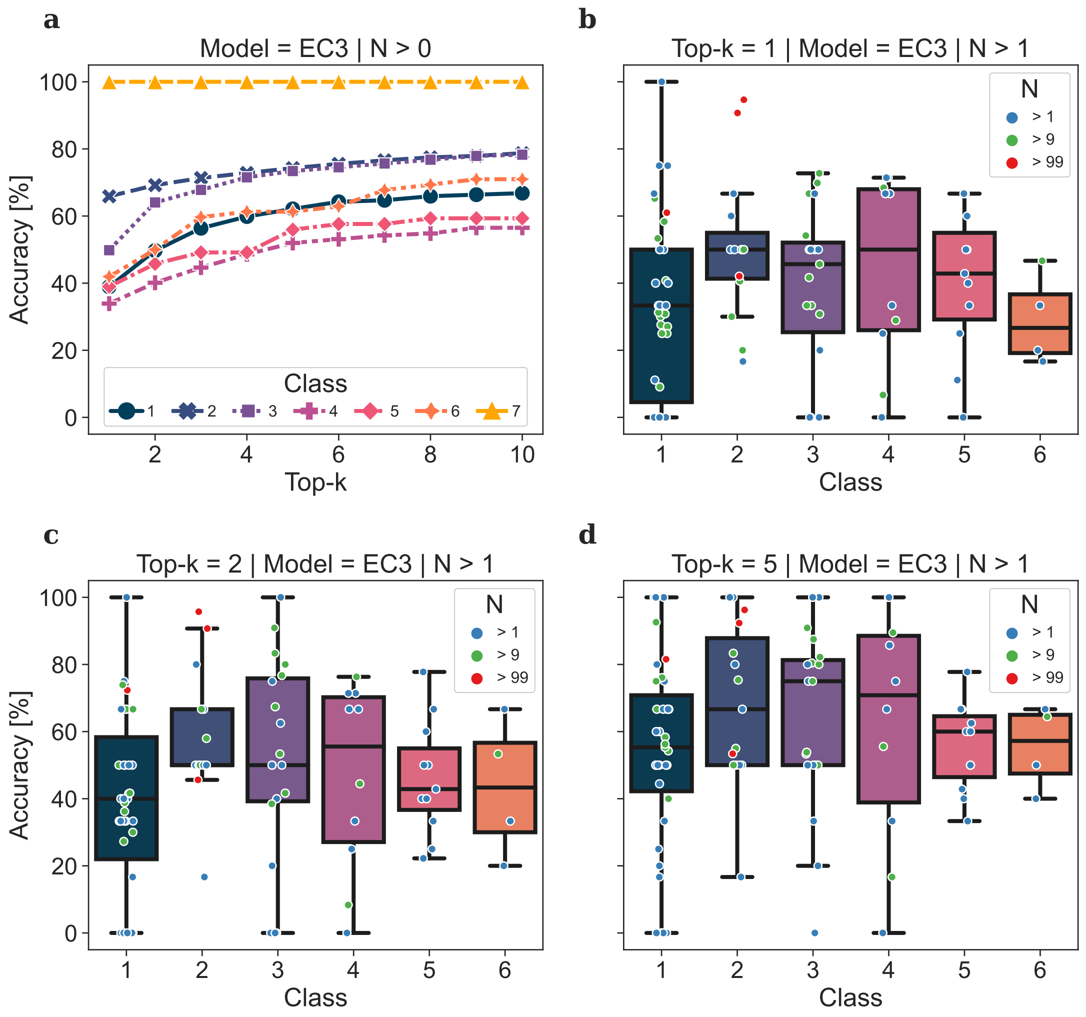

Supplementary Figure 5: Class-wise accuracy for the forward model trained on token scheme EC3 with **stereochemistry information removed**. (**a**) The top-k prediction accuracy for each class. The accuracy of (**b**) top-1, (**c**) top-2, and (**d**) top-5 predictions shown in detail. Each dot represents an EC-level 3 category with a number of test samples > 1. The EC-level 3 subclasses are further stratified by test sample size N. Removing all information related to stereochemistry leads to an increase in overall accuracy from 49.6% to 55%. With the highest increase among isomerase-catylsed reactions (class 5) of 18.6% to 40%.

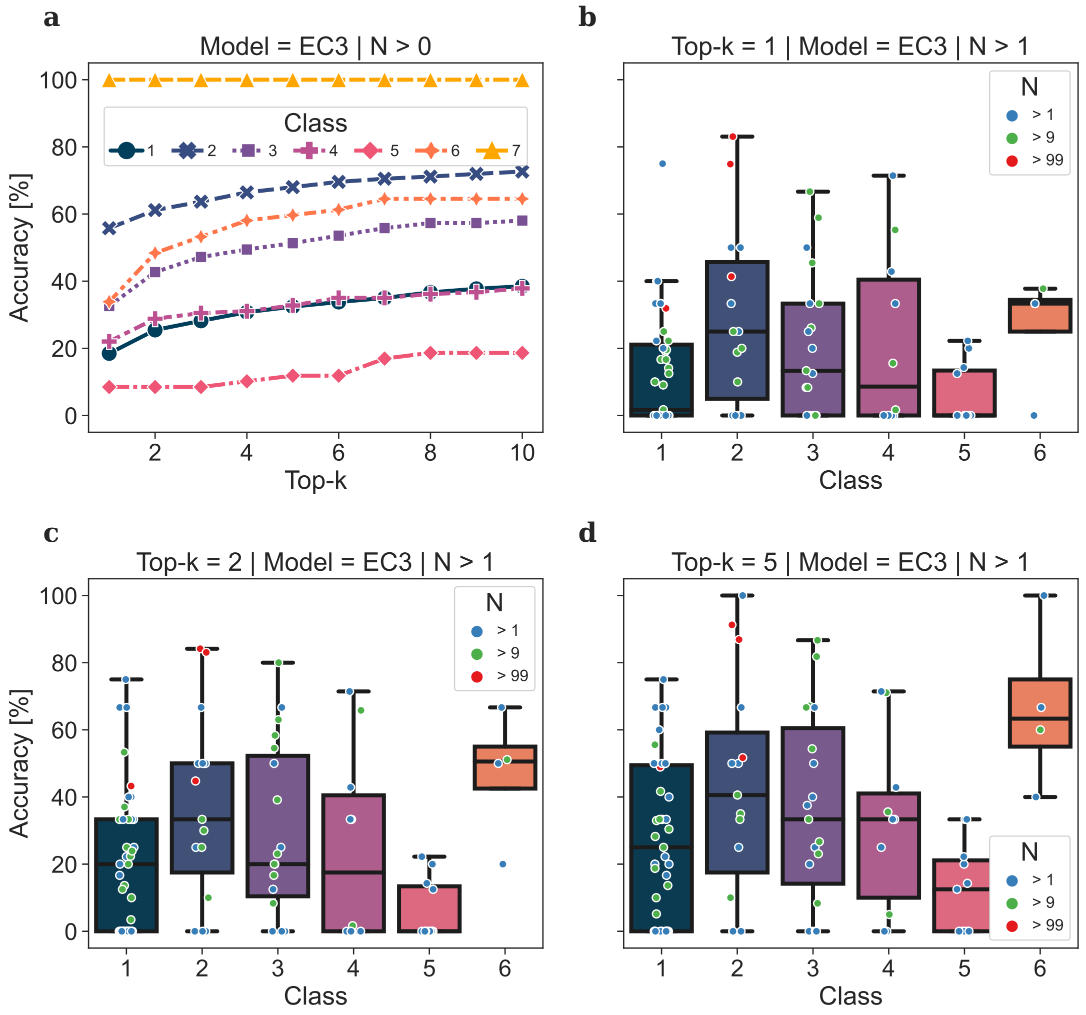

Supplementary Figure 6: Class-wise accuracy for the forward model trained on token scheme EC3 with EC numbers **randomized within classes**. (**a**) The top-k prediction accuracy for each class. The accuracy of (**b**) top-1, (**c**) top-2, and (**d**) top-5 predictions shown in detail. Each dot represents an EC-level 3 category with a number of test samples > 1. The EC-level 3 subclasses are further stratified by test sample size N.

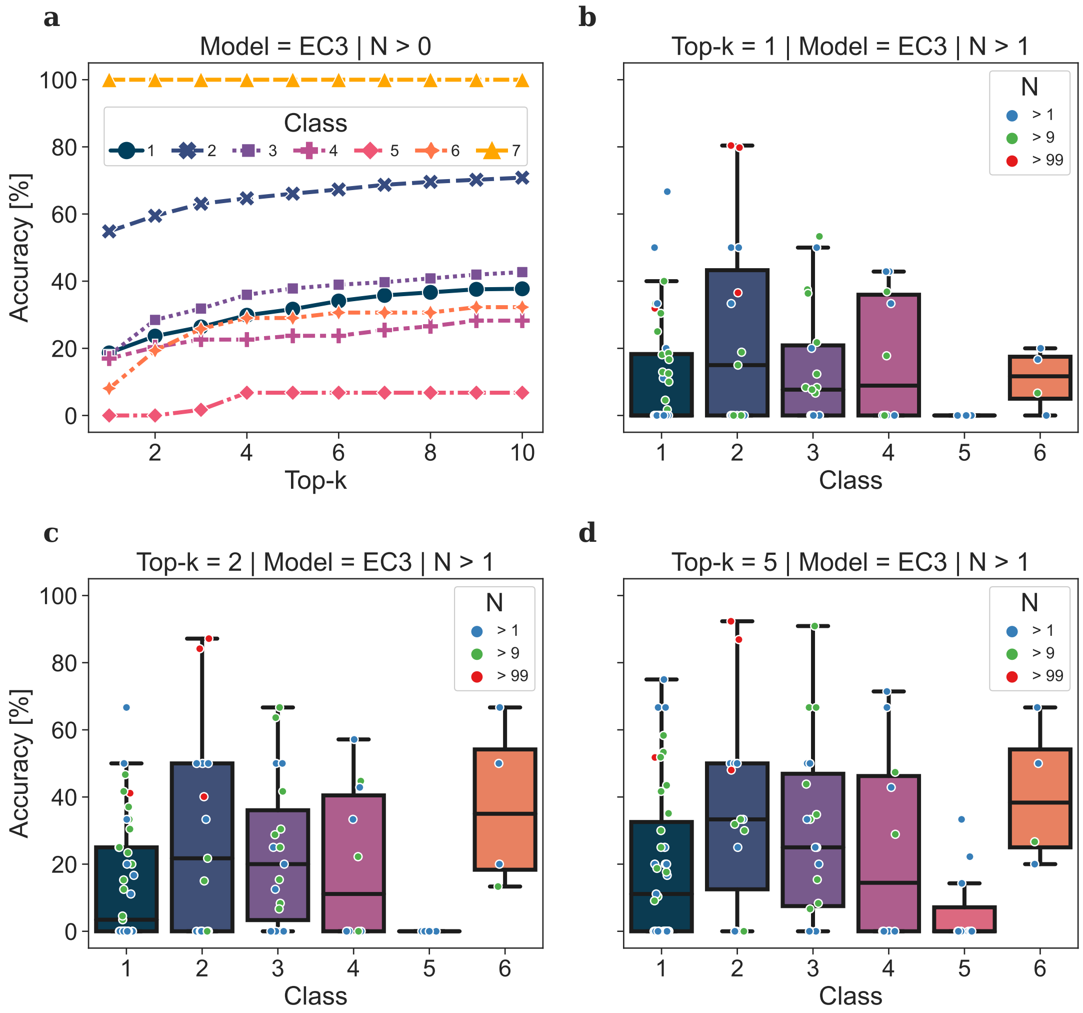

Supplementary Figure 7: Class-wise accuracy for the forward model trained on token scheme EC3 with EC **numbers randomized across classes**. (**a**) The top-k prediction accuracy for each class. The accuracy of (**b**) top-1, (**c**) top-2, and (**d**) top-5 predictions shown in detail. Each dot represents an EC-level 3 category with a number of test samples > 1. The EC-level 3 subclasses are further stratified by test sample size N.

Class	Top-1 Accuracy [%]		
	Non-Randomized	Randomized within Class	Randomized
Oxidoreductases (1)	28.0	18.5	18.6
Transferases (2)	64.4	55.8	54.8
Hydrolases (3)	39.7	32.6	18.0
Lysases (4)	28.8	22.0	16.9
Isomerases (5)	18.6	8.5	0.0
Ligases (6)	32.3	33.9	8.1
Translocases (7)	100.0	100.0	100.0
Overall	49.6	41.3	38.3

Supplementary Table 8: Forward model accuracies with non-randomized and randomized EC numbers.

1 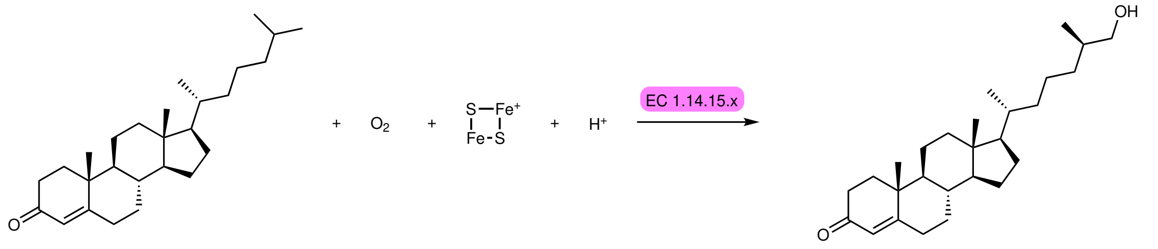

2 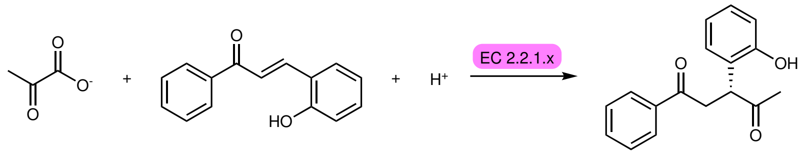

3 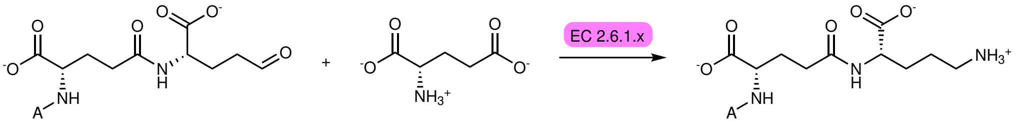

4 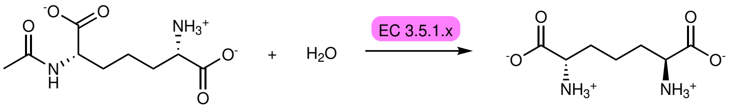

5 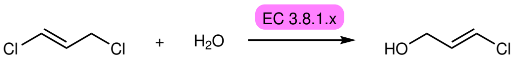

6 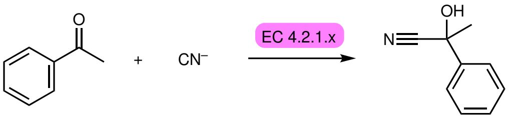

7 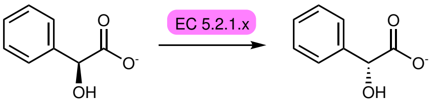

8 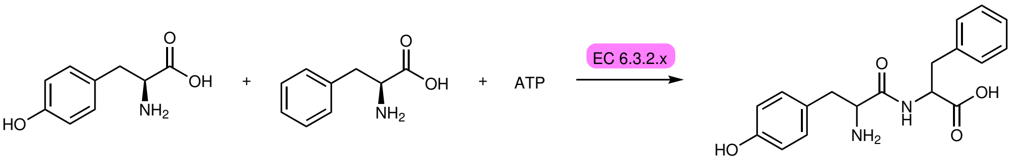

Supplementary Figure 8: Examples of successful forward predictions. The reactions are catalysed by (1) an oxydoreductase with reduced iron-sulfur protein as one donor, and incorporation of one atom of oxygen, (2) aldehyde transferase, (3) an acetylornithine transaminase, (4) a *N*-acetyldiaminopimelate deacetylase, (5) a haloalkane dehalogenase, (6) an (*R*)-mandelonitrile lyase, (7) a mandelate racemase, and (8) an L-alanine-L-anticapsin ligase.

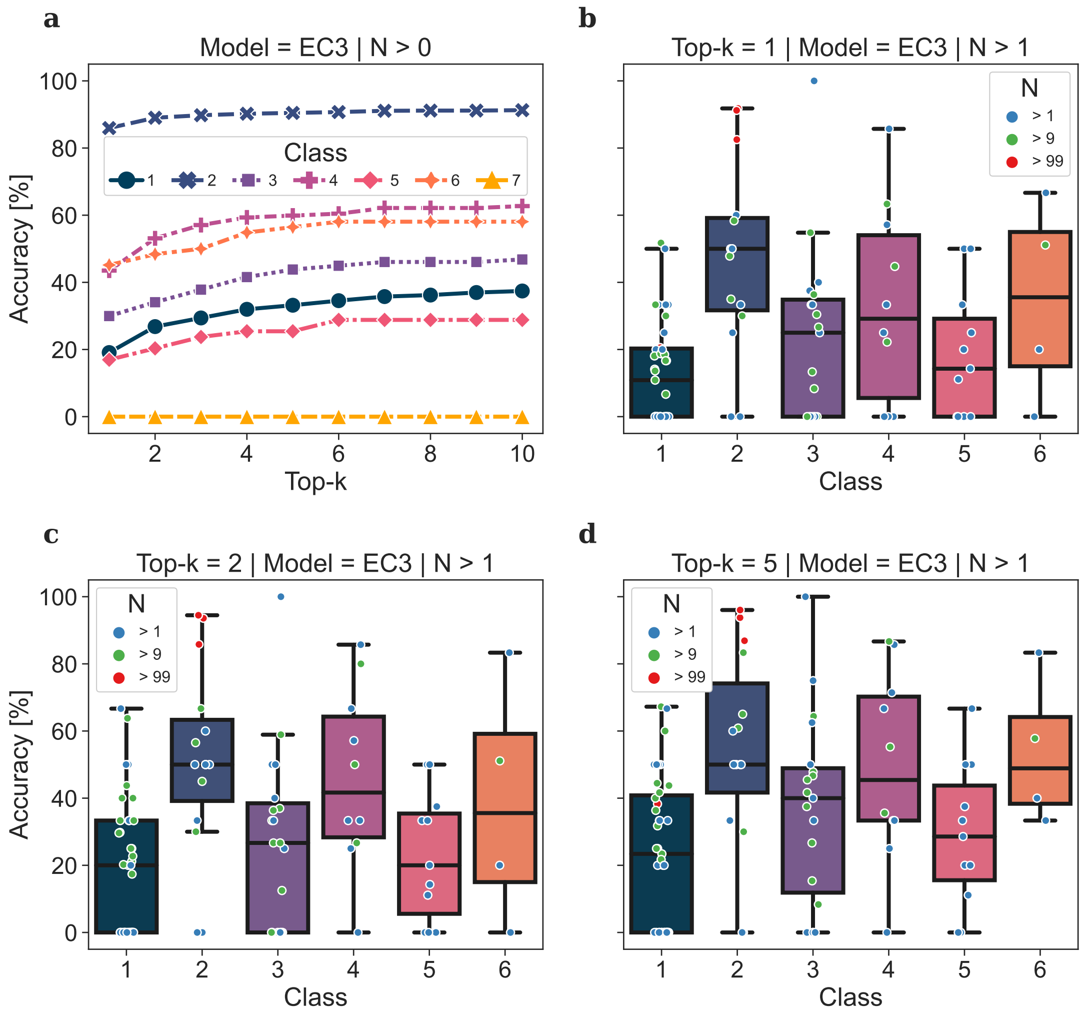

Supplementary Figure 9: Class-wise accuracy for the backward model trained on EC3. (**a**) The top-k prediction accuracies for each class (corresponding to EC-level 1) show significant differences among classes caused by the number of available samples per EC-level 3 category. The accuracy of (**b**) top-1, (**c**) top-2, and (**d**) top-5 predictions per EC-level 3 category. Each dot represents an EC-level 3 category coloured by the number of test samples N. Large EC-level 3 subclasses (red) greatly influence the performance of predicting transferase-catalysed reaction (class 2) outcomes. Oxidoreductase-catalysed reactions (class 1) are distributed among many EC-level 3 subclasses, causing a lower performance compared to other classes with fewer samples overall.

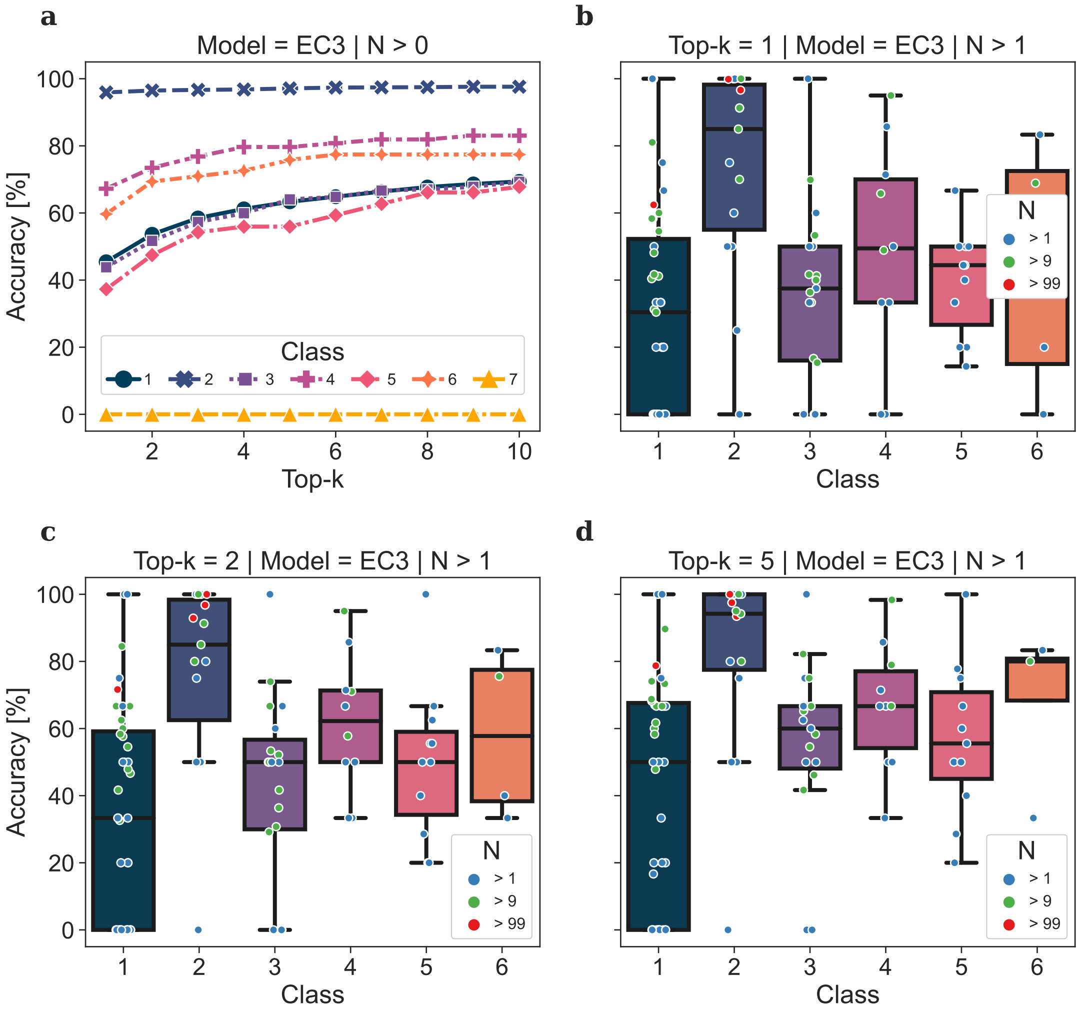

Supplementary Figure 10: Class-wise accuracy for the backward model trained on token scheme EC3 predicting the **EC number only**. (**a**) The top-k prediction accuracy for each class. The accuracy of (**b**) top-1, (**c**) top-2, and (**d**) top-5 predictions shown in detail. Each dot represents an EC-level 3 category with a number of test samples > 1. The EC-level 3 subclasses are further stratified by test sample size N. Given the high number of subclasses for oxidoreductases (class 1) on EC-level 3, it's relatively performance is in line with previous assumptions.

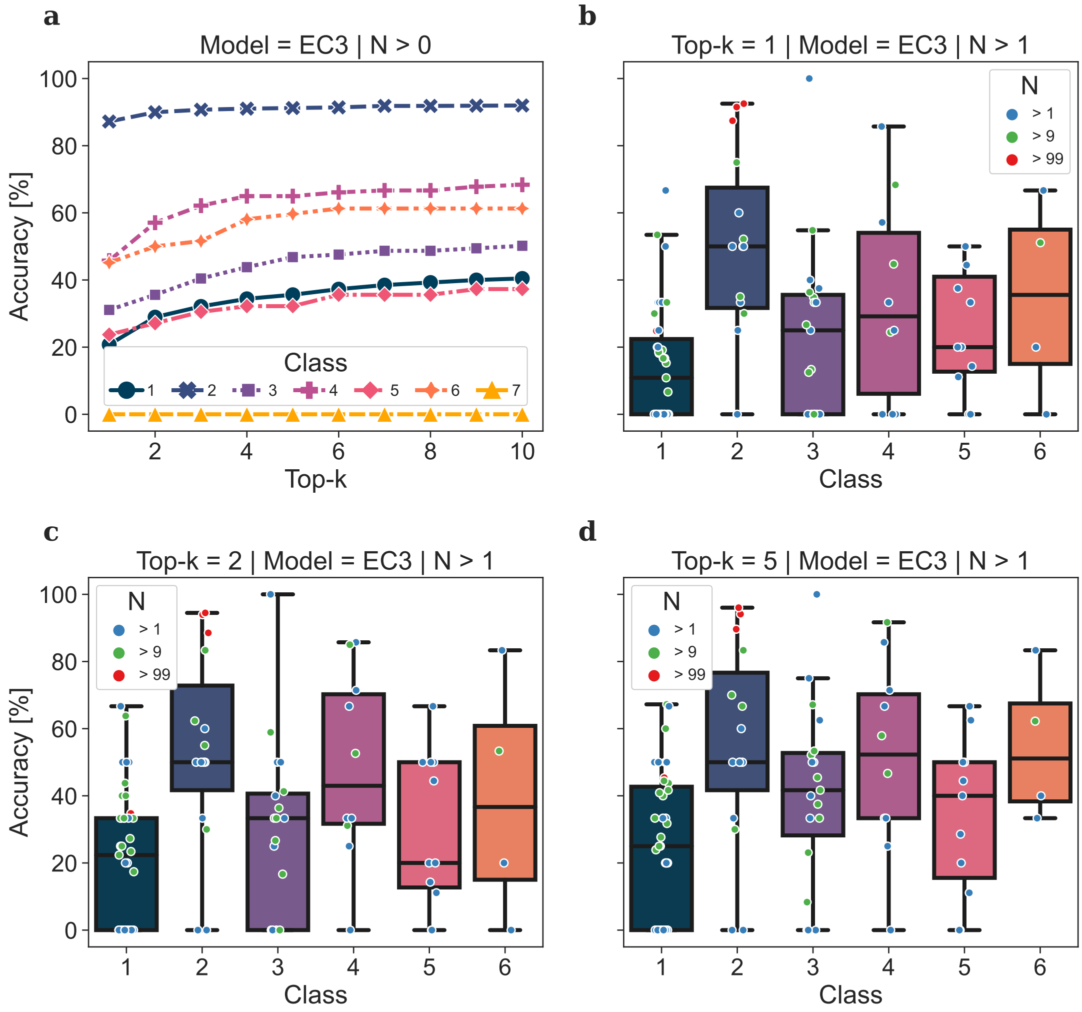

Supplementary Figure 11: Class-wise accuracy for the backward model trained on token scheme EC3 with **stereochemistry information removed**. (**a**) The top-k prediction accuracy for each class. The accuracy of (**b**) top-1, (**c**) top-2, and (**d**) top-5 predictions shown in detail. Each dot represents an EC-level 3 category with a number of test samples > 1. The EC-level 3 subclasses are further stratified by test sample size N.

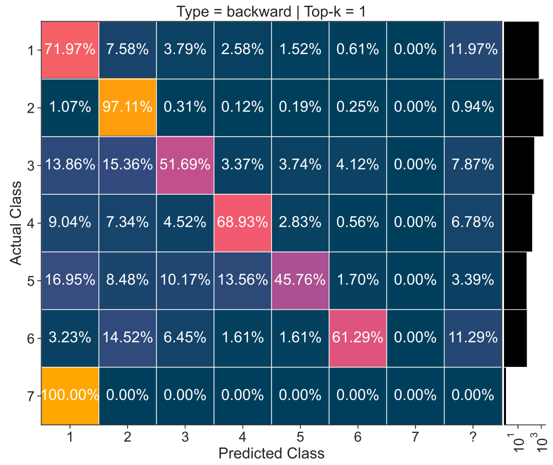

Supplementary Figure 12: The confusion matrix based on predicted EC numbers by the backward model. The bars to the right of the plot show the number of samples per class.

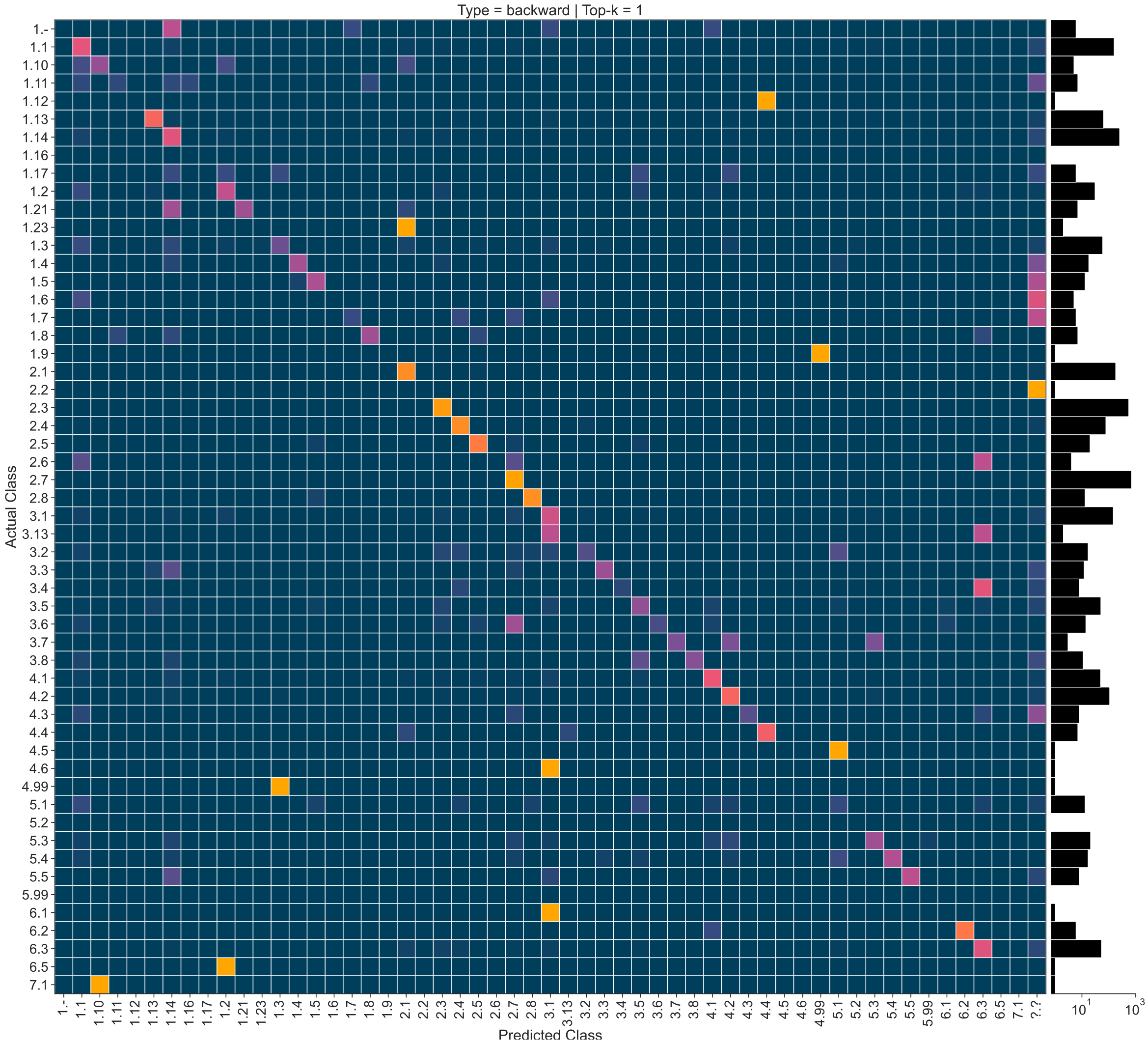

Supplementary Figure 13: The confusion matrix based on predicted EC numbers by the backward model for EC-level 2. The bars right of the plot show the number of samples per EC-level 2 category. Comparing the sample sizes with the respective accuracies shows the established pattern of subclasses with high sample count having also higher accuracy.

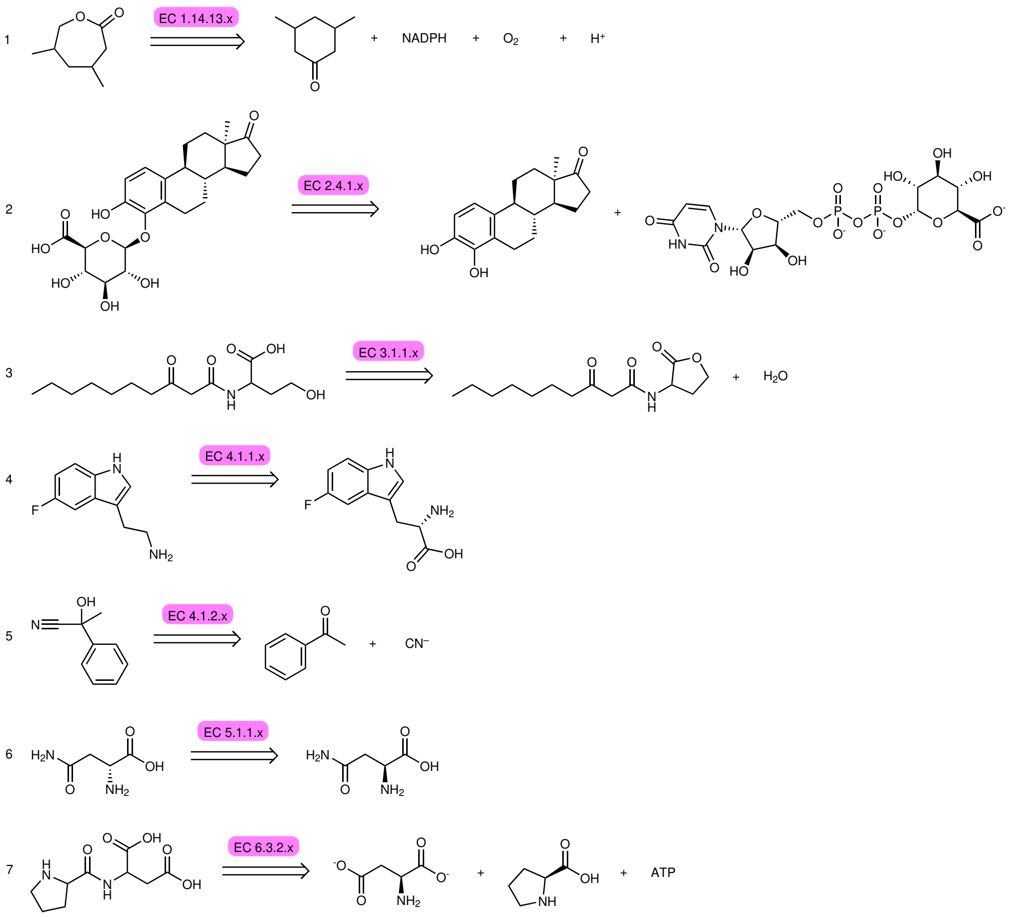

Supplementary Figure 14: Successful backwards predictions. The reactions are catalysed by (1) a cyclohexanone monooxygenase, (2) a glucuronosyltransferase, (3) a quorum-quenching *N*-acyl-homoserine lactonase, (4) an aromatic-L-amino-acid decarboxylase, (5) an aldehyde-lyase, (6) an asparagine racemase, and (7) a peptide synthase.

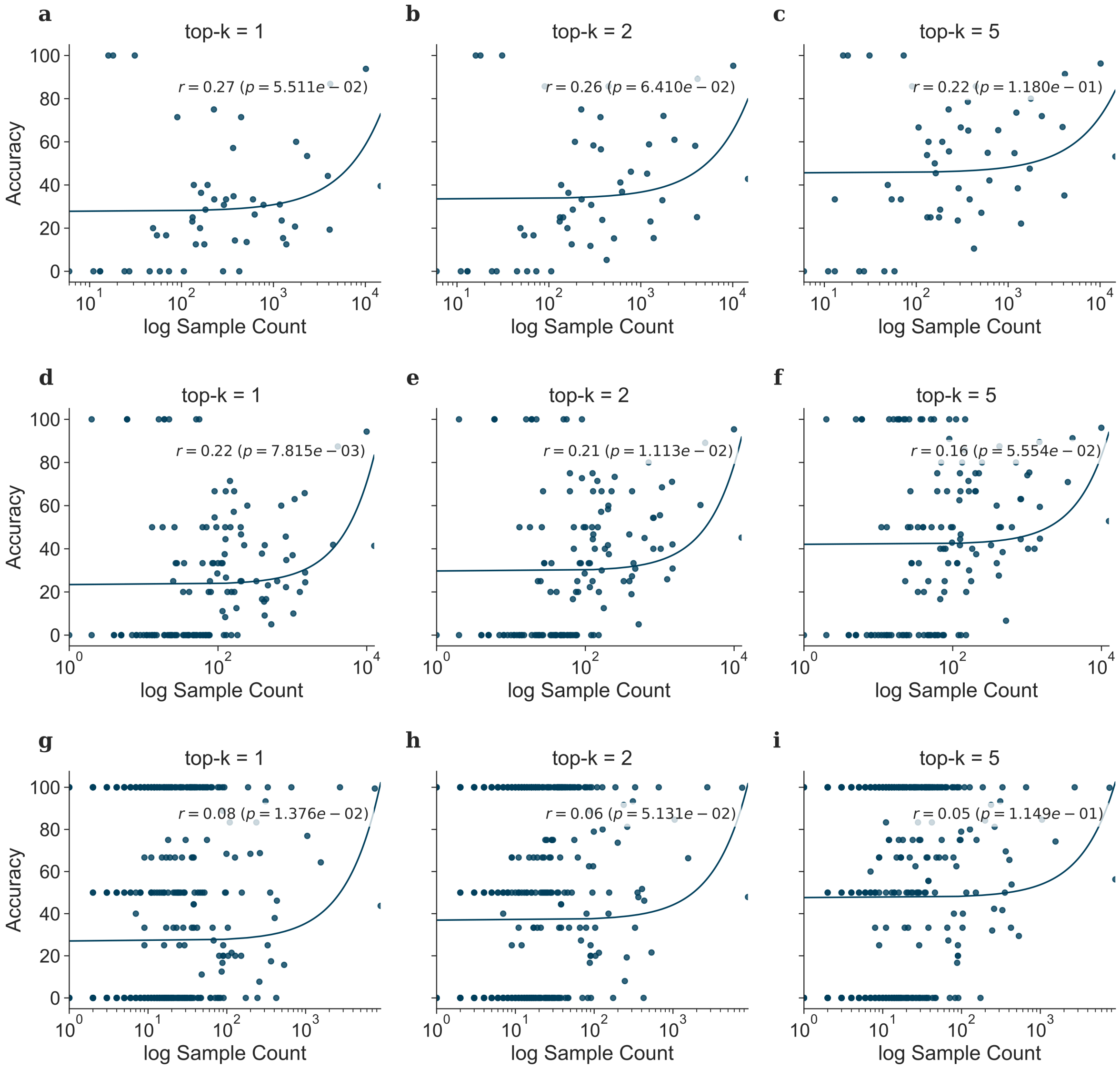

Supplementary Figure 15: Correlation between forward prediction accuracy and sample count in EC2 (**a**, **b**), EC3 (**c**, **d**), and EC4 (**e**, **f**). We observe a significant correlation between sample size in token schemes EC2 and EC3. The trend towards lower correlations in higher EC-level token schemes is caused by a further reduction in test cases due to the selection of unique test products not found in the training sets and the resulting hit-or-miss accuracies appearing as bands at 0 and 100% accuracy, respectively. Increasing k results not only in increasing the accuracy but also in lowering the correlation.

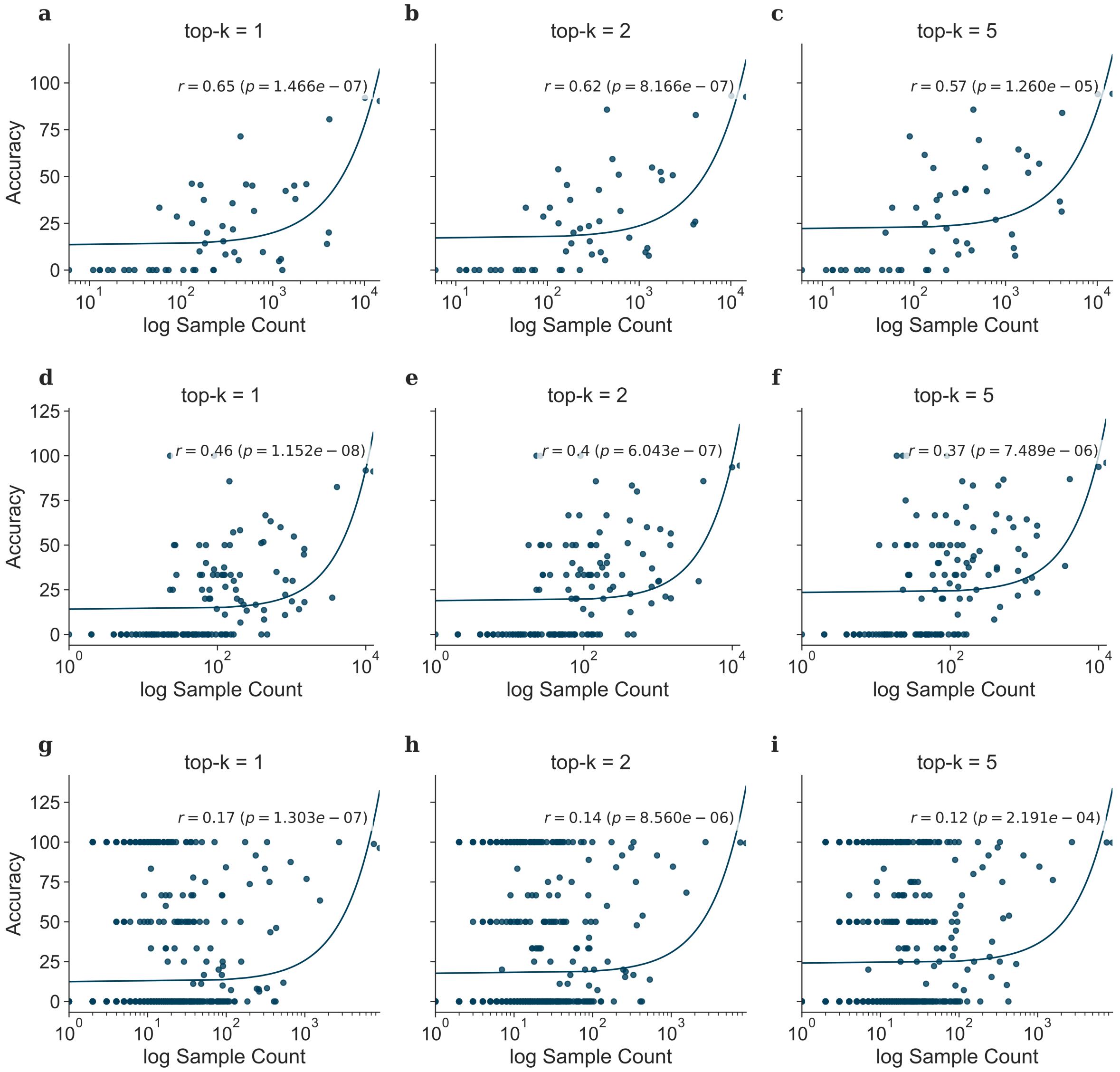

Supplementary Figure 16: Correlation between backward prediction accuracy and sample count in EC2 (**a**, **b**), EC3 (**c**, **d**), and EC4 (**e**, **f**). The trend towards lower correlations in higher EC-level token schemes is caused by a further reduction in test cases due to the selection of unique test products not found in the training sets and the resulting hit-or-miss accuracies appearing as bands at 0 and 100% accuracy, respectively. Increasing k results not only in increasing the accuracy but also in lowering the correlation.

Supplementary Methods

Attention Analysis

The analysis of the patterns in the attention weights of the Molecular Transformer provides insights on the interpretability of these complex models and on potential biases [1]. In the case of reaction SMILES, attention weights have shown to uncover complex reaction information with no supervision, such as atom mappings [2].

In the forward fine-tuned molecular transformer, the connection between the reactants and enzyme components and the products is modelled via self-attention and multi-head attention in the encoder/decoder layers. Since the probability distribution over all prediction candidates is computed based on the current translation state, summarised by the last multi-head attention and the output layer, we focused our analysis on this last part of the decoder by considering only its attention weights.

We used relevant examples from the test set to analyse the patterns emerging from the mean attention over the heads. Using these examples, we investigated attention weights focusing on EC-levels 1-3 of the different heads. We started by analysing all reactions in our test set, focusing at a later stage on the three most frequent enzymatic reaction classes (oxidoreductases, transferases, and hydrolases). Finally, we analysed the correlation between the heads' attention weights to inspect redundancy.

For EC-level analysis, we filtered weights greater than a *noise* threshold. The threshold was set to $\frac{1}{N}$, where N indicates the number of tokens in the input. The value was determined by considering a baseline where each output token uniformly attends all the input tokens, i.e., no specific focus. By masking certain values, we have an appropriate metric to evaluate attention focus. If a token received weights lower than or equal to the threshold, its value was automatically excluded from contributing to the mean calculation. For the correlation analysis, we randomly selected 20 reactions for each class from which we extracted the corresponding head weights. For each reaction, we computed pairwise Pearson correlations [3] between the heads' flattened attention matrices. The correlation matrices for each reaction were aggregated by averaging the Fisher-transformed [4] correlation values. The resulting averaged correlation matrix was then derived by anti-transforming the values using a hyperbolic tangent.

We analyzed the attention patterns across all reactions (see Supplementary Figure 17) and for the most three representative enzymatic reaction classes: oxidoreductases, transferases and hydrolases (see Supplementary Figure 20).

Specific heads focus their attention on the different levels of the EC token, while others attend the complete enzymatic information, attributing comparable weights to all levels of the token. On average, the heads pay more attention to the first two EC number levels and less to the third, causing levels 1 and 2 of the token to be primarily responsible for forward reaction prediction. The comparison of the mean attention for oxidoreductases, transferases and hydrolases reactions (see Supplementary Figure 20) reveals that the model captures variations in enzymatic reactions, focusing on different EC number levels based on the reaction type.

Overall, oxidoreductases exhibit higher values on the enzymatic tokens compared to the others. In contrast, transferases present low values, except for head 3, where the EC number class generally receives higher weights in respect to the average. This explains why transferase data sets can be predicted with only a slight loss of accuracy even when paired with wrong EC numbers. Hydrolases show more variation in attention values, with the highest weight given to the EC-level 2 by head 3. Besides these differences, head 3 always receives the highest attention values, while head 2 receives the lowest of all the reaction classes considered.

In an attempt to capture similarities in attention patterns, we extended our analysis to consider average correlations between the attention heads (see Supplementary Figure 19, details on the correlation analysis can be found in the Methods Section). Attention weights for heads 3, 6 and 7 tends to focus on single tokens (i.e., atoms and EC-levels) and exhibit highly significant correlation values ($\rho_{3,6} = 0.78$, $\rho_{3,7} = 0.65$, $\rho_{6,7} = 0.66$), providing the inherent mapping between tokens/atoms in the reactants and the ones in product. Heads 2 and 4, which tend to focus on the structurally larger group of tokens, e.g., representing branches, show a weakly positive correlation ($\rho_{2,4} = 0.33$). This suggests that the two heads are capturing distinct aspects of the enzymatic reactions while attending similar token lengths. The remaining heads are uncorrelated, highlighting the existence of more complex attention patterns captured by the model.

Supplementary Figure 18 shows a few representative examples of enzymatic reactions and the attention relationship between the EC token levels and the tokens of the product. In all examples, the EC tokens are related to the centre of the enzymatic reaction. Example (a) shows how level 3 of the EC token

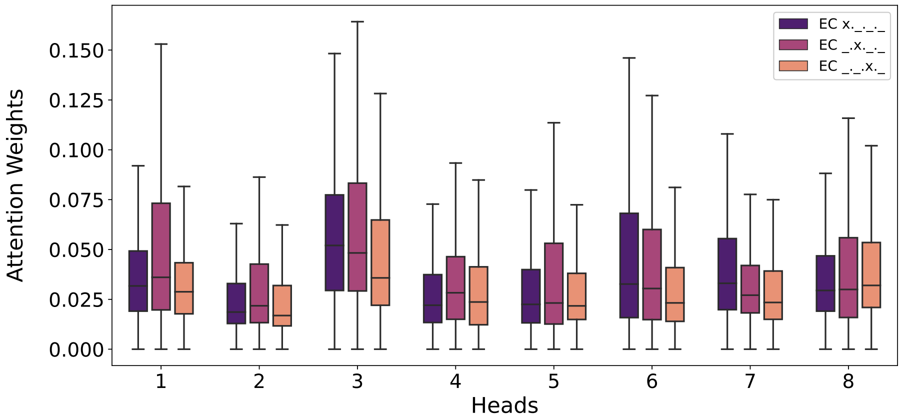

Supplementary Figure 17: Average attention received EC-level 1-3 tokens for each head in the last decoder layer of the forward model considering all reactions in the test set. Although some heads focus on EC-level 3, the majority focuses on EC-levels 1 and 2 stressing their importance in the prediction of the enzymatic reaction outcome. The consistently high values computed for head 3 suggest its importance in the prediction.

focuses on key features of the enzymatic reaction: the centre subject to nucleophilic substitution and the token related to the configurational information. Example (b) reveals the connection between the EC token and the centre of the nucleophilic addition as well as the introduced nucleophile. Finally, example (c) reveals the connection between the EC token and the stereochemical centre undergoing inversion of configuration. The analysis of the attention weights confirms the capacity of the forward Molecular Transformer to use the EC token for discerning the enzymatic reaction centre while capturing enzymatic reaction rules.

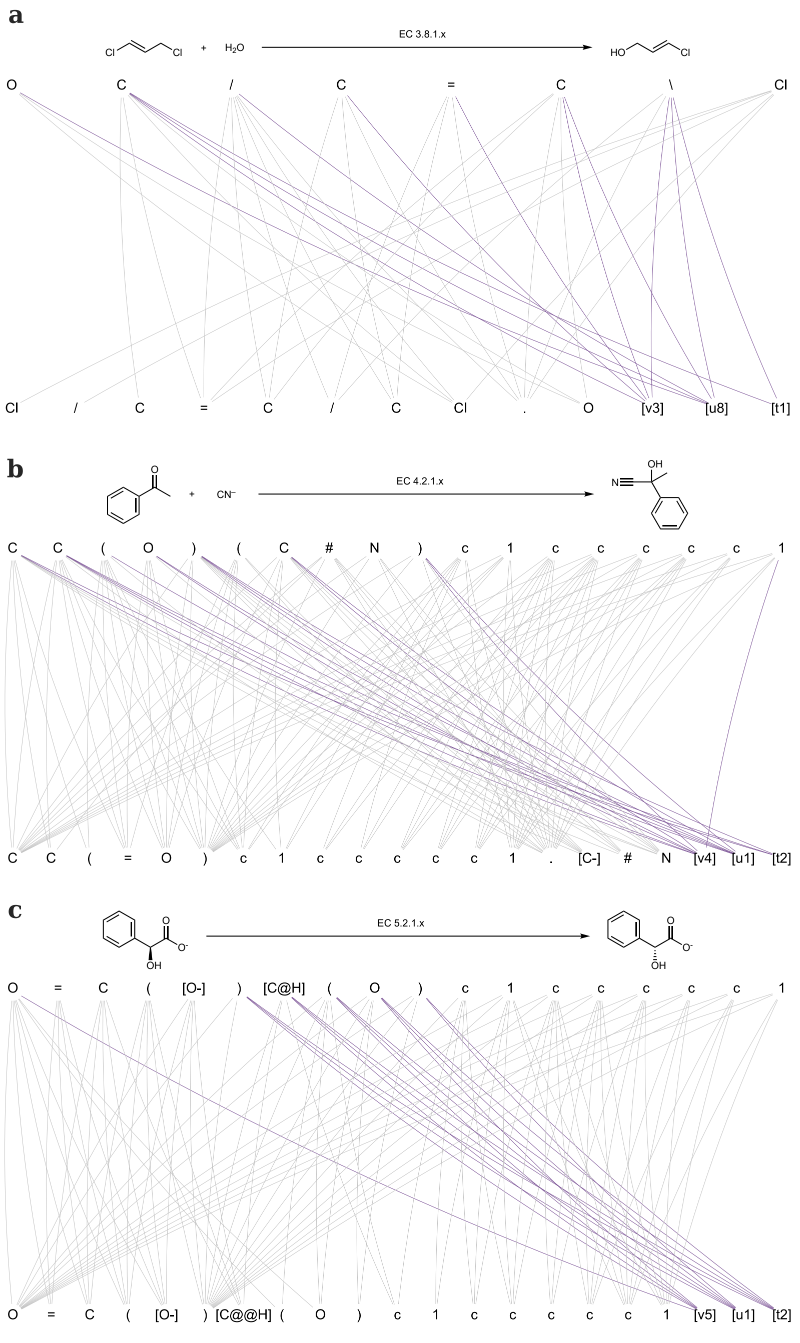

Supplementary Figure 18: Analysis of the attention weights in the forward prediction models on reactions (5), (6) and (7) from Supplementary Figure 8 ((a), (b) and (c) respectively). For each reaction, the attention mapping between tokens representing EC numbers is highlighted in purple (reactant atom tokens are connected using grey curves). The curve thickness is proportional to the attention weight computed by the forward Molecular Transformer.

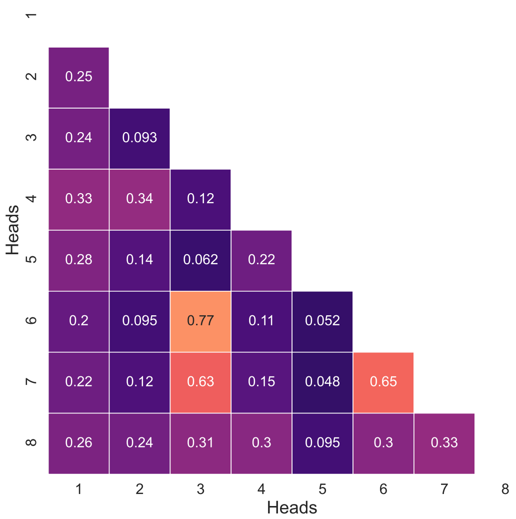

Supplementary Figure 19: The correlation heatmap shows the similarity of the average attention weight received by the heads on the last layer of the decoder of the forward model. Three highly correlated heads (3, 6 and 7) emerge, highlighting preserved patterns among them (attention on single tokens). Other heads, e.g., 2 and 4, show weakly positive correlations highlighting additional preserved patterns (attention on larger groups of tokens). The remaining lower weights indicate the presence of specific patterns that are captured only by specific heads.

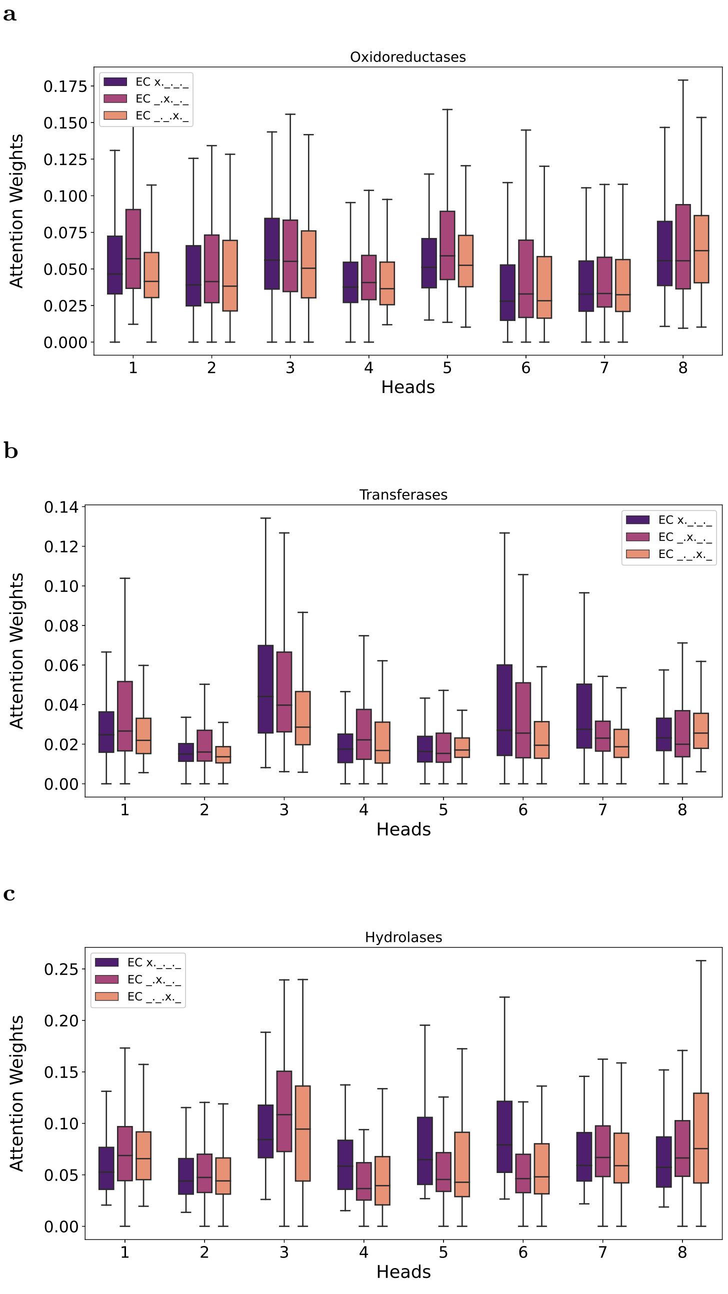

Supplementary Figure 20: Average attention on the EC-level 1-3 tokens for each head using test reactions from the three most represented enzyme classes in the forward model: oxidoreductases (top), transferases (middle), hydrolases (bottom). The difference in the distributions highlight peculiar aspects of each class: oxidoreductases exhibit higher values, transferases relatively low ones, while hydrolases exhibit a more pronounced variability. In general we can appreciate how head 3 shows consistently larger values, unveiling its role in capturing enzymatic information.

Additional Figures

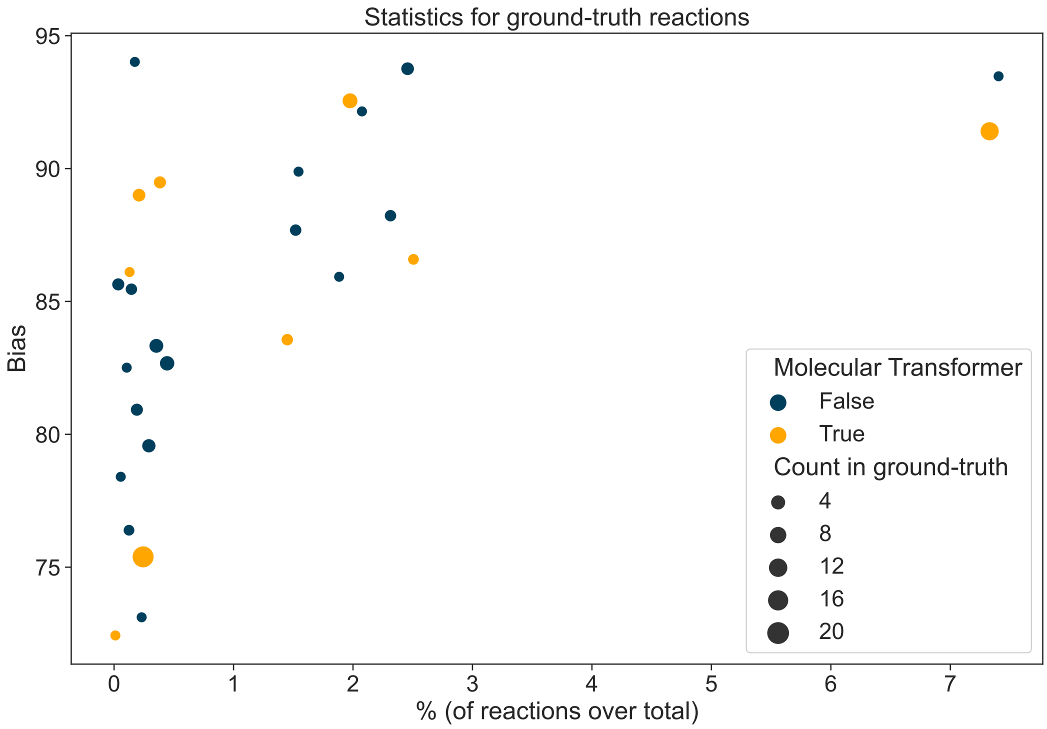

Supplementary Figure 21: Summarized depiction of the most relevant statistics for the curated biocatalysed pathways from Finnigan [5]. In the scatter plot, each enzymatic reaction subclass at EC-level 3 is represented as a point. On the x-axis, we report the percentage of reactions in ECREACT belonging to the class. On the y-axis, we report a biased measure (between 0 and 100) for the EC-level 3 subclass, calculated using the Jensen-Shannon divergence [6] in base 2 between the distribution of EC-level 4 reaction subclasses and a baseline, defined as a uniform distribution of reactions in the EC-level 3 subclass. The bias measure the diversity in the EC-level 3 subclass considered. The point size encodes the number of EC-level 3 reaction subclasses reported in the set of enzymatic reactions from Finnigan [5]. Points are coloured based on the capability of the Molecular Transformer to find a successful route for at least one of the product considered. The depiction shows the high diversity of the reaction subclasses considered in the data sets (bias higher than 70 for all subclasses) and the low sample size for most of the reactions.

Enzymatic reaction

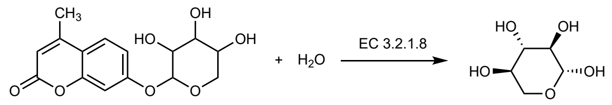

Enzymatic reaction SMILES

`Cc1cc(=O)oc2cc(OC3OCC(O)C(O)C3O)ccc12.O|3.2.1>>O[C@H]1[C@H](O)CO[C@@H](O)[C@@H]1O`

Tokenized reaction

```
C c 1 c c ( = O ) o c 2 c c ( O C 3 O C C ( O ) C ( O ) C 3 O ) c c c 1 2
.
O
|
[v3] [u2] [t1]
>>
O [C@H] 1 [C@H] ( O ) C O [C@@H] ( O ) [C@@H] 1 O
```

Supplementary Figure 22: Step-wise description of the tokenisation process. Starting from an enzymatic reaction (top), a reaction SMILES representation is extracted (middle). The enzymatic reaction SMILES is finally tokenised both at the atom level and at the EC level (bottom).

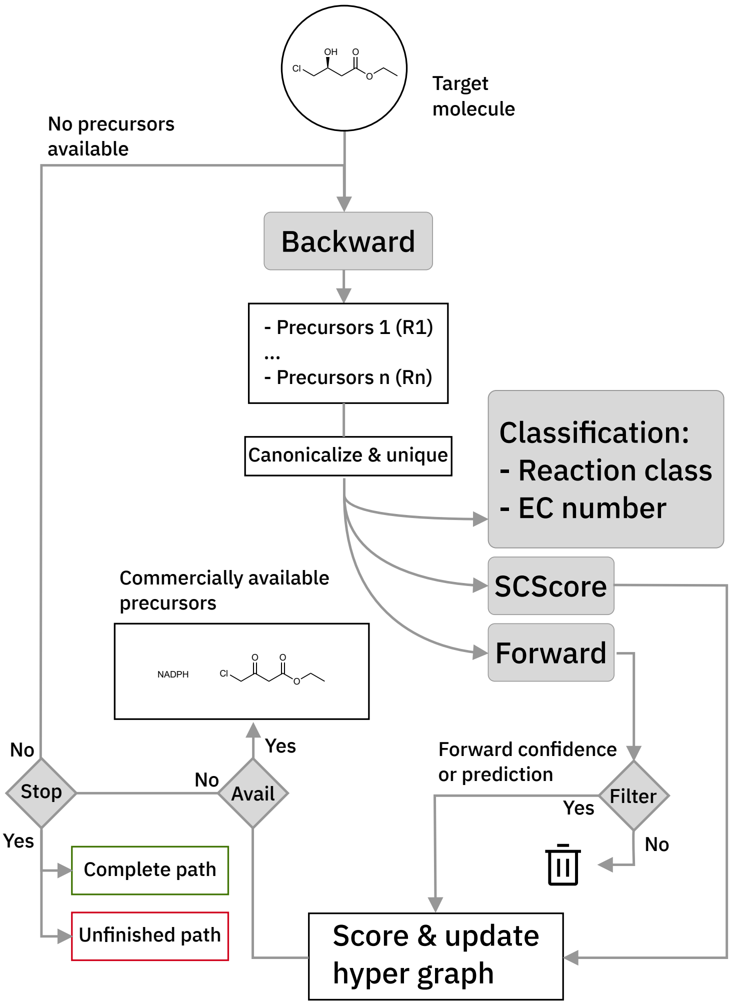

Supplementary Figure 23: Detailed workflow of the retrosynthesis algorithm adapted from [7]. The hypergraph exploration algorithm combining two Molecular Transformer models for forward and backward predictions is extended to handle EC level information at each disconnection predicted by the model encoding it as a reaction class.

Supplementary References

[1] Hoover, Benjamin ; Strobelt, Hendrik ; Gehrmann, Sebastian: exbert: A visual analysis tool to explore learned representations in transformers models. In: *arXiv preprint arXiv:1910.05276* (2019)

[2] Schwaller, Philippe ; Hoover, Benjamin ; Reymond, Jean-Louis ; Strobelt, Hendrik ; Laino, Teodoro: Extraction of organic chemistry grammar from unsupervised learning of chemical reactions. In: *Science Advances* 7 (2021), Nr. 15. – URL `https://advances.sciencemag.org/content/7/15/eabe4166`

[3] Pearson, Karl ; Galton, Francis: VII. Note on regression and inheritance in the case of two parents. In: *Proceedings of the Royal Society of London* 58 (1895), Nr. 347-352, S. 240–242. – URL `https://royalsocietypublishing.org/doi/abs/10.1098/rspl.1895.0041`

[4] Fisher, Ronald A.: Frequency distribution of the values of the correlation coefficient in samples from an indefinitely large population. In: *Biometrika* 10 (1915), Nr. 4, S. 507–521

[5] Finnigan, Will: RetroBioCat database files. (2020), 9. – URL `https://figshare.com/articles/software/RetroBioCat_database_files/12696482`

[6] Fuglede, Bent ; Topsøe, Flemming: Jensen-Shannon divergence and Hubert space embedding. In: *IEEE Int. Symp. Inf. Theory - Proc.*, 2004, S. 31. – ISSN 21578097

[7] Schwaller, Philippe ; Petraglia, Riccardo ; Zullo, Valerio ; Nair, Vishnu H. ; Haeuselmann, Rico A. ; Pisoni, Riccardo ; Bekas, Costas ; Iuliano, Anna ; Laino, Teodoro: Predicting retrosynthetic pathways using transformer-based models and a hyper-graph exploration strategy. In: *Chemical Science* 11 (2020), Nr. 12, S. 3316–3325
